# A predictive signature gene set for discriminating active from latent tuberculosis in Warao Amerindian children

**DOI:** 10.1186/1471-2164-14-74

**Published:** 2013-02-01

**Authors:** Lilly M Verhagen, Aldert Zomer, Mailis Maes, Julian A Villalba, Berenice del Nogal, Marc Eleveld, Sacha AFT van Hijum, Jacobus H de Waard, Peter WM Hermans

**Affiliations:** 1 Laboratory of Pediatric Infectious Diseases, Radboud University Medical Centre, PO Box 9101 (internal post 224), Nijmegen, 6500 HB, The Netherlands; 2 Laboratorio de Tuberculosis, Instituto de Biomedicina, Caracas, Venezuela; 3 Centre for Molecular and Biomolecular Informatics, Nijmegen Centre for Molecular Life Sciences, Radboud University Medical Centre, Nijmegen, The Netherlands; 4 Lovelace Respiratory Research Institute, Albuquerque, USA; 5 Departamento de Pediatría, Hospital de Niños J.M. de los Ríos, Caracas, Venezuela; 6 Facultad de Medicina, Universidad Central de Venezuela, Caracas, Venezuela; 7 NIZO food research, Kluyver Centre for Genomics of Industrial Fermentation, Ede, The Netherlands

**Keywords:** Biomarker, Children, *Mycobacterium tuberculosis*, Transcriptomics

## Abstract

**Background:**

Tuberculosis (TB) continues to cause a high toll of disease and death among children worldwide. The diagnosis of childhood TB is challenged by the paucibacillary nature of the disease and the difficulties in obtaining specimens. Whereas scientific and clinical research efforts to develop novel diagnostic tools have focused on TB in adults, childhood TB has been relatively neglected. Blood transcriptional profiling has improved our understanding of disease pathogenesis of adult TB and may offer future leads for diagnosis and treatment. No studies applying gene expression profiling of children with TB have been published so far.

**Results:**

We identified a 116-gene signature set that showed an average prediction error of 11% for TB vs. latent TB infection (LTBI) and for TB vs. LTBI vs. healthy controls (HC) in our dataset. A minimal gene set of only 9 genes showed the same prediction error of 11% for TB vs. LTBI in our dataset. Furthermore, this minimal set showed a significant discriminatory value for TB vs. LTBI for all previously published adult studies using whole blood gene expression, with average prediction errors between 17% and 23%. In order to identify a robust representative gene set that would perform well in populations of different genetic backgrounds, we selected ten genes that were highly discriminative between TB, LTBI and HC in all literature datasets as well as in our dataset. Functional annotation of these genes highlights a possible role for genes involved in calcium signaling and calcium metabolism as biomarkers for active TB. These ten genes were validated by quantitative real-time polymerase chain reaction in an additional cohort of 54 Warao Amerindian children with LTBI, HC and non-TB pneumonia. Decision tree analysis indicated that five of the ten genes were sufficient to classify 78% of the TB cases correctly with no LTBI subjects wrongly classified as TB (100% specificity).

**Conclusions:**

Our data justify the further exploration of our signature set as biomarkers for potential childhood TB diagnosis. We show that, as the identification of different biomarkers in ethnically distinct cohorts is apparent, it is important to cross-validate newly identified markers in all available cohorts.

## Background

It is estimated that one third of the world’s population is infected with *Mycobacterium tuberculosis* and that each year about nine million people develop tuberculosis (TB), one million (11%) of whom are children under 15 years of age [1]. A unique aspect of TB in children is the rapid progression to disease, typically within the first year following infection, unlike in adults, where TB infection can persist for decades without progression into an active infection [2]. Bacteriological confirmation in the diagnosis of childhood TB is the exception rather than the rule with culture remaining negative in around 70% of cases with probable TB [3]. Using blood transcriptional profiling, several signature gene sets have been identified in adult cohorts from South Africa, The Gambia and The United Kingdom [4-6]. However, a significant overlap was shown with a biomarker set for sarcoidosis, suggesting the need for more specific biomarker sets [7]. To statistically verify differential expression between active TB, latent TB infection (LTBI) and healthy controls (HC) different methods have been used, varying from statistical tests [4,6] to prediction models using the k-nearest neighbours algorithm [4]. Correlation analysis, a method selecting genes that are correlated with a single differentially expressed gene, was used to identify a biomarker set in a Gambian cohort [5]. No studies applying gene expression profiling of children with TB have been published, and it is unknown whether the existing signature gene sets are applicable to childhood cohorts.

In Venezuela, a high TB incidence rate (3190 per 100,000) has been reported in Warao Amerindian children living in the Orinoco Delta in northeastern Venezuela [8]. In this study, we identified new gene signatures in childhood TB by comparing gene expression profiles of Warao Amerindian children with TB, LTBI and HC. We validated the identified gene signatures from this study in an independent cohort of children with LTBI, HC or non-TB pneumonia. Furthermore, we estimated the predictive value of our gene signatures in previously performed adult studies and we compared the discriminatory power of the literature signature gene sets with our gene set.

## Results

### Identification of signature genes

**Figure 1:**
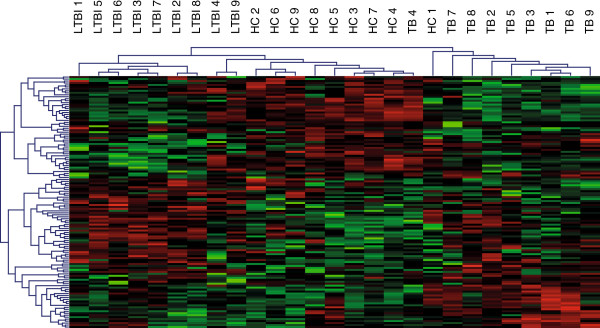
Unsupervised hierarchical cluster analysis of the 116 gene profile.

Genome-wide transcription profiles in whole blood from 9 TB patients, 9 LTBI and 9 HC were generated using Affymetrix exon arrays comprising approximately one million probes, which are mapped to 22011 unique features (Affymetrix core gene set). General characteristics of the study subjects are given in Table 1. Detailed information of the study subjects is given in Additional file 1: Table S1. Random forest analyses were performed to find the signature gene sets used to interrogate whether donors within this study could be divided into distinct groups based on their gene expression profiles. Irrelevant genes were removed from the signature set using the random forest-based local importance measure as described in PhenoLink [9]. A total of 21798 genes were removed in the initial step and the classification or out of bag (OOB) error decreased substantially from 70% to 22%. Next, genes contributing to the correct classification of at least three samples of the same class were selected resulting in a removal of a total of 97 genes and a decrease of the OOB error to approximately 11%. The reduced dataset consisting of 116 genes allowed separation of the three classes with class errors of 11%, 22%, and 0% for the respective classes of TB, LTBI and HC (Table 2). Unsupervised hierarchical cluster analysis of this 116 gene profile showed that all 27 individuals could be successfully clustered into three groups, and each group matched to the corresponding grouping of TB, LTBI and HC (Figure 1). Functional annotation of the 116 genes revealed that genes in the categories of cell proliferation, cell death, phosphorylation and calcium binding were enriched. A full list of enriched gene sets analyzed by the online DAVID tool [10] is provided as supplemental information (Additional file 2: Table S2).

**Table 1:** Characteristics of children with TB, LTBI and HC in which microarray analyses were performed

		**TB**	**LTBI**	**HC**
**Number of donors**		**9**	**9**	**9**
Characteristics				
Age	Mean (SD)	7.8 (5.0)	8.9 (4.5)	7.2 (3.5)
	Range	1.1 – 14.5	2.2 – 14.6	1.3 – 11.5
Gender	Female	7	3	7
	Male	2	6	2

**Table 2:** Class errors of the 116 signature gene set

	**Active TB**	**Latent TB**	**Healthy controls**	**Class error (%)**	**OOB**
Active	8	0	1	11.1	11.1
Latent	0	7	2	22.2	22.2
Healthy	0	0	9	0.0	0.0

### Validation of signature gene sets in independent sample sets

In order to confirm the value of our identified gene set in comparison with existing signature gene sets in all available cohorts that used whole blood gene expression [4-6], we performed random forest classification of the datasets with all signature gene sets to distinguish TB from LTBI and to distinguish TB, LTBI and HC. The average prediction error for the classification of TB and LTBI patients identified by Berry *et al.*[4] using our 116-gene signature set was 20.1% (Table 3). The average error for the two sets of Maertzdorf *et al.*[5,6] was respectively 26.5% and 19.1% (Table 3). Comparison of the other literature sets with each other showed similar average prediction errors when one set was used to discriminate individuals in the other set. However, while our 116-gene set showed a good predictive value for both the childhood cohort described in this study as well as for the adult cohorts identified in other studies, the gene sets identified in those adult studies did not discriminate the children included in our study adequately (average prediction error for TB vs. LTBI 33-50% (Table 3)). As Berry *et al.* provided demographic characteristics of the subjects included in their study [4], we were able to examine whether these demographic characteristics were related to the chance that a subject with TB was wrongly classified as LTBI using our 116 gene set. Multivariable linear regression analysis showed that age and gender were not significantly associated with wrong classification of TB subjects. In contrast, TB patients from South Africa were significantly more often wrongly classified as LTBI than TB patients from London (beta coefficient corrected for age and gender = 0.339, 95% CI 0.213 – 0.465).

**Table 3:** Performance of signature gene sets: cross prediction matrix showing prediction errors

**Class comparison**	**Signature gene set**	**This study**				**Berry *et al. *[**[4]**]**		**Maertzdorf *et al. *[**[6]**]**	
	**Study**	**116 gene set**	**Minimal TB-LTBI set**	**Minimal TB-LTBI-HC set**	**Robust 10 gene q-PCR set**	**86 gene set**	**393 gene set**	**11 gene set**	**5 gene set**
TB vs. LTBI	Berry *et al.*[4]	20.1	19.4	nd^a^	nd	12.5	11.1	13.0	16.3
	Maertzdorf *et al.*[5]	26.5	22.5	nd	nd	19.4	24.5	22.5	21.4
	Maertzdorf *et al.*[6]	19.1	16.9	nd	nd	11.3	10.1	10.2	10.1
	This study	11.1	11.1	nd	nd	50.0	50.0	33.0	50.0
**average prediction error**		**19.2**	**17.5**	nd	nd	**23.3**	**23.9**	**19.7**	**24.5**
TB vs. LTBI vs. HC	Berry *et al.*[4]	27.6	nd	23.9	34.9	20.1	14.1	27.3	30.4
	Maertzdorf *et al.*[5]	48.6	nd	50.3	52.5	47.4	48.6	50.0	41.8
	Maertzdorf *et al.*[6]	25.6	nd	25.6	48.9	17.8	21.3	26.2	32.8
	This study	11.1	nd	14.8	14.8	66.7	74.1	74.1	70.4
		**28.2**	nd	**28.7**	**37.8**	**38.0**	**39.5**	**44.4**	**43.8**
TB vs. LTBI vs. HC vs. other disease	Berry *et al.*[4]	37.4	nd	nd	nd	27.6	18.7	28.5	37.2

Columns represent selected gene biomarker sets in the literature sets as well as in our dataset. Rows represent the studies on which the gene biomarker sets displayed in the columns were tested.

^a^ = not determined.

### Identification of the minimal discriminatory signature gene sets

A minimal discriminatory gene set to discriminate between TB and LTBI was identified by variable selection random forest (VarSelRF) [11]. This procedure produced four genes for the set described in this manuscript, three genes for the dataset described by Berry *et al.*[4], three genes for the dataset described by Maertzdorf *et al.*[5] and two genes for the other dataset described by Maertzdorf *et al.*[6] that are required to distinguish TB from LTBI samples (Additional file 3: Table S3). After removal of the overlaps, *i.e.* the genes that were selected in more than one dataset, nine genes remained (Additional file 3: Table S3). Similarly, a minimal gene set to distinguish TB from LTBI and from HC was identified. In this comparison, six genes were identified for the set described in this manuscript and respectively 35, three, and 25 genes were identified for the three other datasets [4-6] (Additional file 3: Table S3). After removal of the overlaps, 42 genes remained (Additional file 3: Table S3). Performance of these minimal sets was comparable to performance of the 116 gene set (Table 3). However, as only a few genes included in the minimal sets were present in more than one of the datasets, the selected minimal sets seem to be a summary of four small sets that perform well on each of the included datasets rather than a robust representative set that would perform well in ethnically different populations.

### Identification and validation of the optimal signature gene set

Because of potential problems with the extrapolation of the minimal gene signature datasets to other populations due to overfitting of these gene sets on the source datasets, we selected ten genes from the random forest signature 116-gene set that were highly discriminative between the three groups of TB, LTBI and HC in our set of Warao Amerindian children. Furthermore, the ten selected genes were consistently selected in the bootstrapping procedure implemented in VarSelfRF from the set of Berry *et al.* (individuals from The United Kingdom and South Africa, including the individuals suffering from other inflammatory diseases [4]) and from the two sets of Maertzdorf *et al.* (individuals from The Gambia [5] and South Africa [6]). Additional file 3: Table S3 shows the variable frequencies estimated by the bootstrapping procedure per probe per dataset. The selection of ten genes out of these 116 genes consisted of CHRM2, AMPH, SNX17, PIGC, TAS2R46 (downregulated in TB vs. LTBI) and HBD, GLDC, ACOT7, S100P and STYXL1 (upregulated in TB vs. LTBI). These ten selected genes had the highest variable frequency in .632+ bootstrapped runs of the 116 discriminatory gene set in all cohorts, meaning that they were most frequently present in the trees of the random forest analyses performed (Additional file 3: Table S3). Their possible role in TB, lung disease or inflammatory processes is displayed in Table 4. While the minimal set was a mere combination of genes that had a good predictive value for active TB in each of the source databases, the robust ten gene set represents a set of genes that perform well in the discrimination of active TB from LTBI and HC in all datasets used for its composition and probably also as in future cohorts from other study sites. The overlap between the minimal nine gene set for TB vs. LTBI and the robust ten gene set consisted of three genes (HBD, CHRM2 and GLDC) and the overlap between the minimal 42 gene set for TB vs. LTBI vs. HC and the robust ten gene set consisted of seven genes (HBD, CHRM2, GLDC, ACOT7, SNX17, TAS2R46 and PIGC).

**Table 4:** Set of 10 signature genes with their role in TB, lung disease or inflammatory processes

**Gene symbol**	**Gene name**	**Function**	**Possible role in TB, lung disease or inflammatory processes**
CHRM2	Cholinergic muscarine 2 receptor	cAMP regulation on airway smooth muscle.	· Loss of muscarine receptor function is associated with airway hyperreactivity [12].
AMPH	Amphiphysin	Phagocytosis, clathrin-mediated endocytosis in alveolar macrophages [13].	· Clathrin-mediated endocytosis in the lungs plays an important role in mediating the internalization of human rhinovirus and influenza A virus [14,15].
			· Inhibition of clathrin-mediated endocytosis led to inhibition of lipopolysaccharide (LPS) internalization and cytokine/chemokine release from macrophages stimulated by LPS [16].
SNX17	Sorting nexin 17	Intracellular binding protein for the adhesion molecule P-selectin [17].	· P-selectin is important in the early phase of cell migration in TB infection and increased P-selectin serum levels are found in TB patients [18].
PIGC	Phosphatidylinositol glycan anchor biosynthesis class C	Biosynthesis of glycosylphosphatidylinositol [19].	· Incorporation of the mycobacterial cell wall component lipoarabinomannan (LAM) into the macrophage cell membrane, a process that is dependent on successful insertion of a glycosylphosphatidylinositol anchor, is one of the key virulence factors for *M. tuberculosis*[20].
S100P	S100 calcium binding protein P	Calcium-binding protein involved in intracellular and extracellular calcium sensing and signal transduction [21].	· *M. tuberculosis*-mediated inhibition of a cytosolic rise in calcium is one of the essential steps in phagosome maturation [22].
TAS2R46	Taste receptor type 2 member 46	Regulation of ciliary beat frequency through modulation of intracellular calcium concentration [23].	· *M. tuberculosis*-mediated inhibition of a cytosolic rise in calcium is one of the essential steps in phagosome maturation [22]. Decreased expression of TAS2R receptors has been shown to lead to a decrease in intracellular calcium concentration [23].
			· TAS2Rs are expressed on human airway smooth muscle where they cause bronchodilation through a localized calcium response [24].
STYXL1	Serine/threonine/tyrosine interacting-like1	Inhibition of formation of stress granules.	· Stress granules are host RNA cytoplasmic granules formed in response to infections by a pathway involving phosphorylation of the translation initiation factor eIF2α [25].
HBD	Hemoglobin delta	Encodes for the delta globin chain of HbA2.	· Involved in oxygen transport from the lung to the peripheral tissues.
GLDC	Glycine dehydrogenase (decarboxylating)	Metabolic enzyme promoting cellular transformation.	· Altered GLDC expression has been correlated with survival time in lung cancer patients [26].
ACOT7	Acyl-CoA thioesterase 7	Expressed in macrophages, plays a role in inflammation through production of arachidonic acid.	· The molecular and cellular functions of ACOT7 have identified the enzyme as a candidate drug target in inflammatory diseases [27].

**Figure 2:**
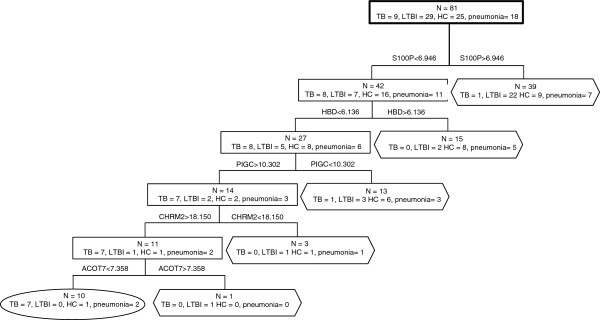
**Decision tree analysis to differentiate TB from LTBI, HC and non-TB pneumonia.** The combination of S100P, HBD, PIGC, CHRM2 and ACOT7 provides the best discrimination between TB, LTBI, HC and non-TB pneumonia. The sensitivity and specificity of this five-gene panel was 78% and 96% respectively. 94% of the children were correctly classified. Rectangle: internal nodes; Oval and hexagon: terminal nodes showing the number finally determined as TB and non-TB (LTBI, HC or non-TB pneumonia).

For validation of the ten genes included in the robust ten gene set, we carried out quantitative real-time polymerase chain reaction (qRT-PCR) studies using the same samples as used for the microarray experiment. Additionally, we tested our identified set of ten genes in 54 additional samples (20 LTBI, 16 HC, 18 non-TB pneumonia) from Warao Amerindian children aged 1 to 15 years. Furthermore, from three children with TB a recovery sample taken five months after initiation of anti-TB treatment was tested with qRT-PCR. Analysis of the qRT-PCR data showed that S100P (*p* = 0.004), GLDC (*p* = 0.016) and HBD (*p* = 0.027) significantly discriminated TB from LTBI while PIGC (*p* = 0.007), SNX17 (*p* = 0.019), TAS2R46 (*p* = 0.017) and HBD (*p* = 0.007) significantly discriminated TB cases from HC. TB cases were separated from non-TB pneumonia cases based on expression of SNX17 (*p* = 0.027) and HBD (*p* = 0.006). Active TB cases were separated from all other groups (*i.e.* LTBI, HC and non-TB pneumonia) based on expression of PIGC (*p* = 0.045), GLDC (*p* = 0.044) and HBD (*p* = 0.025). The values of area under receiver operating characteristic (ROC) curve (AUC) of these genes are shown in Table 5. The quantitative results of qRT-PCR analyses are shown in Additional file 4: Figure S1 and Additional file 5: Table S4. Decision tree analysis indicated that five genes (S100P, HBD, PIGC, CHRM2 and ACOT7) were sufficient to classify 78% of the TB cases correctly with no false-positives among the children with LTBI (100% specificity). Among the HC and non-TB pneumonia cases, false positive rates were 4% and 11% respectively (Figure 2). Interestingly, following this decision tree, the follow-up samples that were taken from three of the nine TB patients when they had received five months of anti-TB treatment were no longer classified as TB while the samples taken on inclusion of these patients were correctly classified as TB.

**Table 5:** Receiver operating characteristic analysis of selected genes

**Gene**	**Down- or upregulation in TB**	**TB (n = 9) vs. LTBI (n = 29)**				**TB (n = 9) vs. HC (n = 25)**			**TB (n = 9) vs. pneumonia (n = 18)**		
		**AUC**	**p-value**	**% Sens**	**% Spec**	**AUC**	**p-value**	**% Spec**	**AUC**	**p-value**	**% Spec**
ACOT7	Upregulation	0.70	0.073	67	86	0.63	0.24	76	0.67	0.15	72
AMPH	Downregulation	0.55	0.86	56	52	0.60	0.91	76	0.56	1.00	61
CHRM2	Downregulation	0.63	0.17	56	62	0.62	0.33	46	0.52	0.87	44
GLDC	Upregulation	0.78	0.016	67	79	0.64	0.19	73	0.66	0.13	72
HBD	Upregulation	0.79	0.027	67	93	0.77	<0.01	78	0.79	<0.01	83
PIGC	Downregulation	0.62	0.29	55	76	0.80	<0.01	89	0.73	0.076	94
S100P	Upregulation	0.80	<0.01	89	76	0.58	0.77	35	0.64	0.24	39
SNX17	Downregulation	0.54	0.67	56	65	0.74	0.019	86	0.71	0.027	83
STYXL1	Upregulation	0.58	0.67	56	65	0.60	0.34	30	0.62	0.27	61
TAS2R46	Downregulation	0.65	0.31	67	72	0.76	0.017	84	0.73	0.071	78

## Discussion

Although peripheral blood transcriptional signatures discriminating between TB, LTBI and HC subjects have been identified in adult studies [4-6], concerns about the specificity of these signature sets have been raised [7]. Furthermore, the performance of these signature sets in children, that show high rates of progressive tuberculosis due to immaturity of the immune response, has not been investigated so far. In this study, we identified a 116 signature gene set that discriminated TB from LTBI and HC with class errors of 11%, 22%, and 0% for the respective classes of TB, LTBI and HC (Table 2). While this 116 gene signature set also showed a good discriminative value between TB and LTBI in adults from South Africa, The Gambia and The United Kingdom, signature sets that were identified in those adult cohorts were unable to discriminate TB from LTBI in our childhood cohort (Table 3).

Gene clusters that were enriched in our signature set included genes in the categories of (programmed) cell death and calcium binding (Additional file 2: Table S2). Both the Gambian as well as the South-African study of Maertzdorf *et al.*[5,6] also described enrichment of genes involved in cell death. Other similarities between the functional annotations in the South African study [6] and our study are the enrichment of genes involved in regulation of cell proliferation, regulation of caspase activity and protein kinase activity. Specifically, CD64 was identified as the most powerful discriminating gene seperating TB from LTBI cases in the South African study [6]. As CD64 has also been identified as a marker for general innate immune response activity and sepsis, this marker may not be specific to TB [28]. Berry *et al.* observed that genes downstream of type I interferon-αβ receptor signaling were over-respresented in patients with active TB [4]. However, type I inferferon signaling is also induced in response to respiratory viruses [29] and *Streptococcus pneumoniae*[30], questioning the specificity of genes involved in type I interferon receptor signaling as biomarkers for active TB.

The enrichment of genes involved in calcium signaling in our TB biomarker set has not been described before in adult studies using whole-blood gene expression [4-6], nor in studies based on transcriptional profiling of peripheral blood mononuclear cells (PBMCs) [31,32]. A close relation between abnormal calcium metabolism and radiological extent of disease has been described in pulmonary TB patients [33,34]. Alterations in serum calcium, particularly cases of hypercalcemia, have been observed in adult TB patients [33-35]. Hypercalcemia in pediatric TB patients is an infrequently recognized and poorly understood phenomenon [36]. In lung tissue, several processes related to calcium homeostasis are thought to contribute to *M. tuberculosis* persistence and the aggregation of macrophages in granulomas. Over-production of 1,2-dihydroxyvitamin D3, which plays a traditional role in calcium metabolism, in alveolar macrophages in granulomas has a protective effect against oxidative injuries from the nitric oxide burst from granulomatous macrophages [37-39]. Furthermore, *M. tuberculosis* inhibits a calcium-dependent phagolysosome formation pathway which leads to the prevention of maturation of *M. tuberculosis*-containing phagosomes into phagolysosomes. This process, referred to as the *M. tuberculosis* phagosome maturation arrest, is critical for *M. tuberculosis* persistence in the human host [22]. S100P, which significantly discriminated TB from LTBI in our study children, and TAS2R46, which significantly distinguished TB cases from HC, are genes involved in calcium signaling [21,23,24]. Possibly, altered expression of these genes in TB patients reflects *M. tuberculosis*-mediated changes in calcium metabolism in lung tissue that can be measured in peripheral whole blood.

Although the groups of TB, LTBI and HC were reasonably well age-matched (Table 1), we cannot exclude the possibility that age-dependent differences in immune responses have influenced gene expression profiles. Age-related differences in both innate as well as antigen-specific responses to *M. tuberculosis* are well recognised [40,41]. Alveolar macrophage antimicrobial activity and recruitment of monocytes as well as the production of cytokines and certain aspects of antigen presentation appear to be less efficient in young children. This is particularly true in children younger than one year of age [41]. Therefore, the exclusion of children less than one year of age in our study is likely to have prevented a significant influence of age-related immune differences on gene expression results. Furthermore, the signature set that we identified showed a good discriminative value between TB and LTBI in adults from several regions [4-6]. This is an indication that the genes that were selected in our analysis make up a signature set that performs well in individuals of all ages.

We identified a minimal gene set of 42 genes that was able to separate TB cases from LTBI and HC in all previously described (adult) cohorts [4-6] as well as in our childhood cohort. However, as this minimal set was possibly over-optimized to fit exactly those sets that were used for its composition, this set might not perform well in a newly identified cohort from a different geographic region. As the datasets used for the composition of the minimal set were based on European, African and South American populations the minimal set may not be applicable to individuals from Asia, while this region carries almost two-third of the global TB burden [42]. Furthermore, this signature set could be only indicative of damage to the lung epithelium, similar to what has been described for the overlap of the gene set determined by Berry *et al.*[4] and the biosignature characteristic for sarcoidosis [7]. Therefore, we used bootstrapping procedures to select a robust set of ten genes that had a high discriminative value in our population, in the two populations described by Maertzdorf *et al.*[5,6] and in the comparison between TB, LTBI, HC and other inflammatory and infectious diseases in the dataset of Berry *et al.*[4]. Although this approach probably leads to less overfitting of the selected set towards the source databases used and less overlap with other infectious diseases in comparison with the minimal gene set we identified, the discriminatory power of this ten gene set is less than that of the minimal set (Table 3). Future cohorts can be of help in the reduction of the 116 gene set to a dataset with similar performance in discriminating TB from LTBI, HC and other inflammatory diseases as the minimal gene set without overfitting the dataset to the source datasets.

From the ten gene set, a combination of five (S100P, HBD, PIGC, CHRM2 and ACOT7) could be used in decision tree analysis to differentiate TB from LTBI, HC and non-TB pneumonia with 78% sensitivity and 96% specificity in our dataset (Figure 2). Additionally, the expression profile of children that were treated for TB shifted from an active TB classification (oval in Figure 2) towards a classification as not suffering from active TB (hexagon in Figure 2) at five months post treatment initiation. This indicates that these biomarkers reflect a dynamic response that changes as mycobactericidal activity diminishes.

The discriminatory value of the 116 gene signature set for the classification of cases in the cohort described by Berry and colleagues [4] was significantly better in people from London compared to people from South Africa. An explanation for the greater similarity between our study population with people from London than with people from South Africa comes from population-genetic studies in which a decrease in the level of genetic variation between populations is observed with increasing geographic distance from Africa, consistent with the out-of-Africa spread of human populations [43]. The finding that previously published signature sets based on individuals from South Africa [4,6] do not provide a good discriminatory value between TB, LTBI and HC in The Gambia [5] points towards a high heterozygosity in TB immune response between different African countries. A high-resolution survey of genotype variation based on single-nucleotide polymorphisms, copy-number variants and haplotype analysis of a worldwide sample of 29 populations revealed that the genetic distance between individuals from Asia and Native American or Colombian individuals is significantly less than the genetic distance between Asian and South African populations [44]. Bayesian cluster analysis clustered individuals from East Asia together with Native American or Colombian individuals, indicating their close phylogenetic relationship [44]. Clustering of Native American individuals with Asian individuals based on their genetic similarities was also observed in a recently published quantitative assessment of human genetic variation worldwide [45]. Therefore, we speculate that the applicability of our signature set in Asian populations might be better than the applicability of sets identified in African or European populations.

## Conclusions

This study provides a signature gene set that was demonstrated to be instrumental for the diagnosis of childhood TB. As the identification of different biomarkers in ethnically distinct cohorts is apparent, it is important to cross-validate newly identified markers in all available cohorts. Especially, more childhood cohorts should be investigated as TB diagnosis based on traditional methods is less sensitive and specific in children compared to adults.

## Methods

### Study population

The Warao Amerindians are an indigenous population living in wooden houses raised on stilts along the Orinoco river banks. With a population of approximately 30,000, the Warao people are the second most important Native American group in Venezuela. In this study, 27 HIV-negative children 1 to 15 years of age with TB (n = 9), LTBI (n = 9) and HC (n = 9) were recruited between May 2010 and December 2010. General characteristics of the study subjects are given in Table 1. Detailed information of the study subjects that was recorded on inclusion is given in Additional file 1: Table S1. Tuberculin skin test (TST) and QuantiFERON-TB Gold In-Tube assay (QFT-GIT) were performed in all children. Sputum samples were collected from all children with expectoration and gastric aspirates were taken from all children under 6 years of age. Children with active TB were diagnosed based on culture of *M. tuberculosis* (n = 2) or on the basis of clinical, epidemiological and radiological features (n = 7). The latter group were children with a TST ≥ 10 mm or a positive QFT-GIT result who presented all of: persistent fever > 38°C recorded daily for at least two weeks, persistent cough for more than three weeks, weight loss (> 5% reduction in weight compared with the highest weight recorded in last three months) or failure to thrive (documented crossing of percentile lines in the preceding three months), persistent lethargy or decrease in playfulness/activity reported by the parent and absence of clinical response on broad-spectrum antibiotics. Standard antero-posterior and lateral chest radiographs (CXRs) were taken from all children. Two independent experts, blinded to all clinical information, evaluated the CXRs and documented their findings on a standard report form. Where the two objective experts disagreed, a third expert was consulted and final consensus was achieved. A diagnosis of TB was only made when the CXR was consistent with TB [46] and the child showed a positive clinical response to anti-TB treatment. Children were followed up clinically, radiologically and, in case of a negative TST at inclusion, by means of TST at six and 12 months after inclusion. LTBI was defined as a TST ≥ 10 mm and a positive QFT-GIT with a negative culture result on inclusion in the absence of radiological and clinical evidence of TB disease on inclusion as well as on six and 12 months after inclusion. HC were children with a TST = 0 mm on inclusion and on six and 12 months after inclusion. The HC had a negative QFT-GIT and a negative culture result on inclusion in the absence of radiological and clinical evidence of TB disease on inclusion as well as on six and 12 months after inclusion. TB patients were sampled before initiation of anti-TB treatment. Of three of the nine TB patients, a follow-up sample was taken when the patient had taken anti-TB treatment for five months.

### RNA isolation and microarray procedures

From every child, 2.5 ml of peripheral whole blood was collected in PAXgene RNA tubes (PreAnalytix, Hombrechtikon, Switzerland) and stored at −80°C prior to processing. RNA was isolated using the PAXgene Blood RNA kit (PreAnalytix) following the manufacturer’s protocol. RNA quality was checked using the RNA 6000 Nano Kit on a Bioanalyzer 2100 (Agilent Technologies, Santa Clara, CA). Gene expression data were obtained using GeneChip Human Exon 1.0 ST Arrays (Affymetrix, Santa Clara, CA). The probe preparation and hybridization was done following Affymetrix protocol. Arrays were washed and stained according to the protocol on a GeneChip Fluidics Station 450 (Affymetrix) and scanned on a Genechip Scanner 3000 + autoloader (Affymetrix). Scan results were converted to CEL with the Affymetric scanning software. Spot intensity data were processed with Affymetrix Power Tools (version 1.14-4.1.1) using robust multi-array average (RMA) and plier-gcbg normalization on the core gene set (version HuEx-1 0-st-v2.r2.dt1.hg18). Gene expression values were log2-transformed and differentially expressed genes were identified based on log2 fold changes (M-values). P-values were calculated with a Bayes-regularized one-way ANOVA [47] followed by multiple testing correction of the p-values (q-values) according to the method of Storey and Tibshirani [48]. Microarray data have been deposited in the GEO database under accession GSE41055 (http://www.ncbi.nlm.nih.gov/geo/query/acc.cgi?token=ltmhxwsmskeyyte&acc=GSE41055).

### Random forest based identification of TB biomarker genes

A random forest classification was performed to identify signature genes for discrimination of TB, LTBI and HC. This classification model, consisting of 5000 decision trees trained on random subsets of samples and variables was trained based on log2 gene expression data as a function of individuals belonging to either TB, LTBI or HC classes. Irrelevant genes were removed using the random forest-based local importance measure as described in PhenoLink [9] where genes with a negative or neutral contribution of correctly classifying the samples were removed. This process was repeated until fewer than three genes could be removed per iteration. Next, genes were selected provided that they had a contribution to the correct classification of at least three samples of the same class. Again, this process was repeated until fewer than three genes were removed. The resulting set was used for classification and determination of the classification error, both as OOB error and as average error, on all gene expression sets.

### Literature datasets

Literature datasets describing gene expression studies of TB, LTBI, HC and other infectious diseases were obtained from GEO (http://nar.oxfordjournals.org/content/39/suppl_1/D1005.full), accession numbers GSE19491 [4], GSE19492 [4], GSE28623 [5] and GSE25534 [6]. For GSE19491, GSE19492 and GSE28623 available normalized data were used, while for GSE25534 the 2 dye array data was normalized using RMA in Arraystar (DNAStar, Madison, WI) to allow inter-slide comparison of gene expression data. First, the TB biomarker set identified in this manuscript was applied to the literature cohorts [4-6] and the biomarker sets identified in the literature cohorts were applied to each other for estimation of the predictive value of each identified set in the other populations. Next, to determine the minimal discriminatory gene sets based on the dataset described in this study as well as on the three previously published datasets [4-6], expression data from probes corresponding with the TB biomarker set determined in this manuscript were subjected to VarSelRF, as described by Diaz-Uriarte *et al.*[11], which progressively eliminates genes with the lowest random forest-based local importance measure until no further improvements in the OOB error rate are reported. This procedure is designed to identify small, non-redundant sets of genes that have good predictive performance. However, over fitting of the selected minimum discriminatory gene sets on the source databases used could lead to a poor performance of these minimal sets when applying them to classify other patient populations. Therefore, .632+ bootstrapping procedures were performed to select genes with a high discriminative power in all datasets. Bootstrapping repeatedly analyzes subsamples rather than subsets of the data. As each subsample is a random sample with replacement from the full sample, this procedure performs well in the selection of classifiers even when there is over fitting [49].

To test possible associations between the class probability of being wrongly classified as LTBI (dependent variable) and age, gender and geographical region (independent variables) of TB cases included in the GSE19491/GSE19492 dataset, multivariable linear regression analysis was performed.

### Functional analysis

Enrichment analysis of signature genes was performed using the web-based DAVID bioinformatics tool (http://david.abcc.ncifcrf.gov) [10]. Q*-*values for enriched annnotations were determined by a modified Fisher’s exact test (EASE Score [10]) and corrected for multiple testing by the Benjamini-Hochberg approach.

### Quantitative RT-PCR on microarray and additional samples

Differential expression of several genes was validated by qRT-PCR. cDNA was generated by reverse transcription using Superscript III Reverse Transcriptase and Random Primers (Invitrogen) following manufacturer’s protocol after DNAse treatment of the RNA using TURBO DNA-free (Ambion). GAPDH was chosen as reference gene. qRT-PCR was performed on the 27 microarray samples as well as on a validation cohort (n = 54). These were RNA samples collected from an additional 54 children during the same period and in the same manner as the microarray samples. Of the 54 children of which RNA was collected for qRT-PCR, 20 were diagnosed with LTBI, 16 were HC and 18 children were diagnosed with a radiologically confirmed pneumonia [50], of which six were LTBI and 12 were HC.

ROC methodology was applied to evaluate the discriminatory ability of signature genes. All expression values were normally distributed (Kolmogorov-Smirnov’s test, *p* > 0.05). One-way analysis of variance and unpaired Student’s *t* tests were performed to compare expression values of these genes in children with TB, LTBI, HC and non-TB pneumonia. When the variances across groups were not equal (Levene’s test *p* < 0.05), Welch correction for nonhomogeneity of variance was applied.

### Ethical considerations

The nature and objectives of the study were explained to the parents of children in Spanish or were simultaneously translated to their native language by Spanish-Warao bilingual native interpreters. The study was approved by the ethical committee of the Instituto de Biomedicina, the Regional Health Services, and the Delta Amacuro Indigenous Health Office (Servicio de Atención y Orientación al Indígena). Children were enrolled if their parents or primary caregivers provided written informed consent. Illiterate parents or caregivers signed by means of a thumb print.

## Competing interests

All authors declare that they have no competing interests.

## Authors’ contributions

LV, MM and JV: patient recruitment and sampling; LV and ME: RNA isolation, microarray and qRT-PCR experiments; AZ and SvanH: bioinformatic analyses; JdeW: coordination and funding of field work and sampling logistics; BdelN: advise on patient recruitment and sampling; PH: supervision and funding of microarray, qRT-PCR and bioinformatic analyses; LV, AZ and PH wrote the paper. All authors commented on the manuscript. All authors read and approved the final manuscript.

## Supplementary Material

Additional file 1: Table S1 Detailed characteristics of 27 children with TB, LTBI and HC in which microarray analyses were performed. Click here for file

Additional file 2: Table S2 Enriched gene sets in the 116 gene set. Click here for file

Additional file 3: Table S3 Sheet 1. Minimal discriminatory gene set to discriminate between TB and LTBI. Sheet 2. Minimal discriminatory gene set to discriminate between TB, LTBI and HC. Sheet 3. Variable frequencies estimated by the bootstrapping procedure implemented in VarSelRF (1). Sheet 4. Variable frequencies estimated by the bootstrapping procedure implemented in VarSelRF (2). Click here for file

Additional file 4: Figure S1 qRT-PCR cycle threshold (delta Ct) values in TB, LTBI, HC and non-TB pneumonia subjects for each of the ten signature genes. The delta Ct was calculated as Ct value (number of cycles required for the fluorescent signal to exceed the background level) of the target gene – Ct value of the reference gene (GAPDH). The boxplots show the median delta Ct values and the interquartile ranges. The whiskers represent the highest and lowest values that are not outliers. Dots represent outliers and squares in the boxes indicate mean delta Ct values. The p-values for each gene (in parentheses) are the outcomes of the one-way analyses of variance comparing TB, LTBI, HC and non-TB pneumonia. The asterisks indicate statistically significant differences (*p* < 0.05) between TB and other groups as found by an unpaired Student’s *t* test. Click here for file

Additional file 5: Table S4 qRT-PCR cycle threshold (delta Ct) values in TB, LTBI, HC and non-TB pneumonia subjects for each of the ten signature genes. The delta Ct was calculated as Ct value (number of cycles required for the fluorescent signal to exceed the background level, a lower Delta Ct value indicates a higher expression) of the target gene – Ct value of the reference gene (GAPDH). Click here for file

## References

[B1] World Health Organization (WHO) Guidance for national tuberculosis programmes on the management of tuberculosis in children 2006 Geneva, Switzerland: WHO 24999516

[B2] Newton SM Brent AJ Anderson S Whittaker E Kampmann B Paediatric tuberculosis Lancet Infect Dis 2008 8 498 510 18652996 10.1016/S1473-3099(08)70182-8 PMC2804291

[B3] Zar HJ Hanslo D Apolles P Swingler G Hussey G Induced sputum versus gastric lavage for microbiological confirmation of pulmonary tuberculosis in infants and young children: a prospective study Lancet 2005 365 130 134 15639294 10.1016/S0140-6736(05)17702-2

[B4] Berry MP Graham CM McNab FW Xu Z Bloch SA Oni T Wilkinson KA Banchereau R Skinner J Wilkinson RJ Quinn C Blankenship D Dhawan R Cush JJ Mejias A Ramilo O Kon OM Pascual V Banchereau J Chaussabel D O’Garra A An interferon-inducible neutrophil-driven blood transcriptional signature in human tuberculosis Nature 2010 466 973 977 20725040 10.1038/nature09247 PMC3492754

[B5] Maertzdorf J Ota M Repsilber D Mollenkopf HJ Weiner J Hill PC Kaufmann SH Functional correlations of pathogenesis-driven gene expression signatures in tuberculosis PLoS One 2011 6 e26938 22046420 10.1371/journal.pone.0026938 PMC3203931

[B6] Maertzdorf J Repsilber D Parida SK Stanley K Roberts T Black G Walzl G Kaufmann SH Human gene expression profiles of susceptibility and resistance in tuberculosis Genes Immun 2011 12 15 22 20861863 10.1038/gene.2010.51

[B7] Maertzdorf J Weiner J III Mollenkopf HJ Network T Bauer T Prasse A Muller-Quernheim J Kaufmann SH Common patterns and disease-related signatures in tuberculosis and sarcoidosis Proc Natl Acad Sci USA 2012 109 7853 7858 22547807 10.1073/pnas.1121072109 PMC3356621

[B8] Fernández De Larrea C Fañdino C López D Del Nogal B Rodríguez N Convit J Araujo Z De Waard JH Tuberculosis en menores de 15 años en la población Warao de Venezuela Invest Clin 2002 43 35 48 11921746

[B9] Bayjanov JR Molenaar D Tzeneva V Siezen RJ Van Hijum SA PhenoLink - a web-tool for linking phenotype to omics data for bacteria: application to gene-trait matching for Lactobacillus plantarum strains BMC Genomics 2012 13 170 22559291 10.1186/1471-2164-13-170 PMC3366882

[B10] Da Huang W Sherman BT Lempicki RA Systematic and integrative analysis of large gene lists using DAVID bioinformatics resources Nat Protoc 2009 4 44 57 19131956 10.1038/nprot.2008.211

[B11] Diaz-Uriarte R GeneSrF and varSelRF: a web-based tool and R package for gene selection and classification using random forest BMC Bioinformatics 2007 8 328 17767709 10.1186/1471-2105-8-328 PMC2034606

[B12] Coulson FR Fryer AD Muscarinic acetylcholine receptors and airway diseases Pharmacol Ther 2003 98 59 69 12667888 10.1016/s0163-7258(03)00004-4

[B13] Yamada H Ohashi E Abe T Kusumi N Li SA Yoshida Y Watanabe M Tomizawa K Kashiwakura Y Kumon H Matsui H Takei K Amphiphysin 1 is important for actin polymerization during phagocytosis Mol Biol Cell 2007 18 4669 4680 17855509 10.1091/mbc.E07-04-0296 PMC2043535

[B14] Lau C Wang X Song L North M Wiehler S Proud D Chow CW Syk associates with clathrin and mediates phosphatidylinositol 3-kinase activation during human rhinovirus internalization J Immunol 2008 180 870 880 18178826 10.4049/jimmunol.180.2.870

[B15] Wang H Jiang C Influenza A virus H5N1 entry into host cells is through clathrin-dependent endocytosis Sci China C Life Sci 2009 52 464 469 19471869 10.1007/s11427-009-0061-0 PMC7089391

[B16] Wang Y Yang Y Liu X Wang N Cao H Lu Y Zhou H Zheng J Inhibition of clathrin/dynamin-dependent internalization interferes with LPS-mediated TRAM-TRIF-dependent signaling pathway Cell Immunol 2012 274 121 129 22341560 10.1016/j.cellimm.2011.12.007

[B17] Florian V Schluter T Bohnensack R A new member of the sorting nexin family interacts with the C-terminus of P-selectin Biochem Biophys Res Commun 2001 281 1045 1050 11237770 10.1006/bbrc.2001.4467

[B18] Mukae H Ashitani J Tokojima M Ihi T Kohno S Matsukura S Elevated levels of circulating adhesion molecules in patients with active pulmonary tuberculosis Respirology 2003 8 326 331 12911826 10.1046/j.1440-1843.2003.00471.x

[B19] Watanabe R Inoue N Westfall B Taron CH Orlean P Takeda J Kinoshita T The first step of glycosylphosphatidylinositol biosynthesis is mediated by a complex of PIG-A, PIG-H, PIG-C and GPI1 EMBO J 1998 17 877 885 9463366 10.1093/emboj/17.4.877 PMC1170437

[B20] Welin A Winberg ME Abdalla H Sarndahl E Rasmusson B Stendahl O Lerm M Incorporation of Mycobacterium tuberculosis lipoarabinomannan into macrophage membrane rafts is a prerequisite for the phagosomal maturation block Infect Immun 2008 76 2882 2887 18426888 10.1128/IAI.01549-07 PMC2446741

[B21] Austermann J Nazmi AR Muller-Tidow C Gerke V Characterization of the Ca2+ − regulated ezrin-S100P interaction and its role in tumor cell migration J Biol Chem 2008 283 29331 293340 18725408 10.1074/jbc.M806145200 PMC2662020

[B22] Vergne I Chua J Singh SB Deretic V Cell biology of mycobacterium tuberculosis phagosome Annu Rev Cell Dev Biol 2004 20 367 394 15473845 10.1146/annurev.cellbio.20.010403.114015

[B23] Shah AS Ben-Shahar Y Moninger TO Kline JN Welsh MJ Motile cilia of human airway epithelia are chemosensory Science 2009 325 1131 1134 19628819 10.1126/science.1173869 PMC2894709

[B24] Deshpande DA Wang WC McIlmoyle EL Robinett KS Schillinger RM An SS Sham JS Liggett SB Bitter taste receptors on airway smooth muscle bronchodilate by localized calcium signaling and reverse obstruction Nat Med 2010 16 1299 1304 20972434 10.1038/nm.2237 PMC3066567

[B25] Lindquist ME Lifland AW Utley TJ Santangelo PJ Crowe JE Jr Respiratory syncytial virus induces host RNA stress granules to facilitate viral replication J Virol 2010 84 12274 12284 20844027 10.1128/JVI.00260-10 PMC2976418

[B26] Zhang WC Shyh-Chang N Yang H Rai A Umashankar S Ma S Soh BS Sun LL Tai BC Nga ME Bhakoo KK Jayapal SR Nichane M Yu Q Ahmed DA Tan C Sing WP Tam J Thirugananam A Noghabi MS Pang YH Ang HS Mitchell W Robson P Kaldis P Soo RA Swarup S Lim EH Lim B Glycine decarboxylase activity drives non-small cell lung cancer tumor-initiating cells and tumorigenesis Cell 2012 148 259 272 22225612 10.1016/j.cell.2011.11.050

[B27] Forwood JK Thakur AS Guncar G Marfori M Mouradov D Meng W Robinson J Huber T Kellie S Martin JL Hume DA Kobe B Structural basis for recruitment of tandem hotdog domains in acyl-CoA thioesterase 7 and its role in inflammation Proc Natl Acad Sci USA 2007 104 10382 10387 17563367 10.1073/pnas.0700974104 PMC1965522

[B28] van der Meer W Pickkers P Scott CS van der Hoeven JG Gunnewiek JK Hematological indices, inflammatory markers and neutrophil CD64 expression: comparative trends during experimental human endotoxemia J Endotoxin Res 2007 13 94 100 17621550 10.1177/0968051907079101

[B29] Garcia-Sastre A Biron CA Type 1 interferons and the virus-host relationship: a lesson in detente Science 2006 312 879 882 16690858 10.1126/science.1125676

[B30] Parker D Martin FJ Soong G Harfenist BS Aguilar JL Ratner AJ Fitzgerald KA Schindler C Prince A Streptococcus pneumoniae DNA initiates type I interferon signaling in the respiratory tract MBio 2011 2 e00016 11 21586648 10.1128/mBio.00016-11 PMC3101776

[B31] Jacobsen M Repsilber D Gutschmidt A Neher A Feldmann K Mollenkopf HJ Ziegler A Kaufmann SH Candidate biomarkers for discrimination between infection and disease caused by Mycobacterium tuberculosis J Mol Med (Berl) 2007 85 613 621 17318616 10.1007/s00109-007-0157-6

[B32] Lu C Wu J Wang H Wang S Diao N Wang F Gao Y Chen J Shao L Weng X Zhang Y Zhang W Novel biomarkers distinguishing active tuberculosis from latent infection identified by gene expression profile of peripheral blood mononuclear cells PLoS One 2011 6 e24290 21904626 10.1371/journal.pone.0024290 PMC3164189

[B33] Chan TY Chan CH Shek CC Davies PD Hypercalcemia in active pulmonary tuberculosis and its occurrence in relation to the radiographic extent of disease Southeast Asian J Trop Med Public Health 1992 23 702 704 1298076

[B34] Deniz O Tozkoparan E Yonem A Ciftci F Bozkanat E Cakir E Ozcan O Narin Y Bilgic H Ekiz K Demirci N Low parathormone levels and hypercalcaemia in patients with pulmonary tuberculosis: relation to radiological extent of disease and tuberculin skin test Int J Tuberc Lung Dis 2005 9 317 321 15786897

[B35] Dosumu EA Momoh JA Hypercalcemia in patients with newly diagnosed tuberculosis in Abuja Nigeria. Can Respir J 2006 13 83 87 10.1155/2006/407609 PMC2539012 16550265

[B36] Payne HA Menson E Sharland M Bryant PA Symptomatic hypercalcaemia in paediatric tuberculosis Eur Respir Rev 2011 20 53 56 21357892 10.1183/09059180.00006910 PMC9487729

[B37] Cadranel JL Garabedian M Milleron B Guillozzo H Valeyre D Paillard F Akoun G Hance AJ Vitamin D metabolism by alveolar immune cells in tuberculosis: correlation with calcium metabolism and clinical manifestations Eur Respir J 1994 7 1103 1110 7925880

[B38] Chang JM Kuo MC Kuo HT Hwang SJ Tsai JC Chen HC Lai YH 1-alpha,25-Dihydroxyvitamin D3 regulates inducible nitric oxide synthase messenger RNA expression and nitric oxide release in macrophage-like RAW 264.7 cells J Lab Clin Med 2004 143 14 22 14749681 10.1016/j.lab.2003.08.002

[B39] Carlberg C Campbell MJ Vitamin D receptor signaling mechanisms: Integrated actions of a well-defined transcription factor Steroids 2012 online publication ahead of print 10.1016/j.steroids.2012.10.019 PMC4668715 23178257

[B40] Lewinsohn DA Gennaro ML Scholvinck L Lewinsohn DM Tuberculosis immunology in children: diagnostic and therapeutic challenges and opportunities Int J Tuberc Lung Dis 2004 8 658 674 15137550

[B41] Smith S Jacobs RF Wilson CB Immunobiology of childhood tuberculosis: a window on the ontogeny of cellular immunity J Pediatr 1997 131 16 26 9255187 10.1016/s0022-3476(97)70120-3

[B42] World Health Organization (WHO) Global Tuberculosis Report 2012 2012 Geneva, Switzerland: WHO

[B43] DeGiorgio M Jakobsson M Rosenberg NA Out of Africa: modern human origins special feature: explaining worldwide patterns of human genetic variation using a coalescent-based serial founder model of migration outward from Africa Proc Natl Acad Sci USA 2009 106 16057 16062 19706453 10.1073/pnas.0903341106 PMC2752555

[B44] Jakobsson M Scholz SW Scheet P Gibbs JR VanLiere JM Fung HC Szpiech ZA Degnan JH Wang K Guerreiro R Bras JM Schymick JC Hernandez DG Traynor BJ Simon-Sanchez J Matarin M Britton A van de Leemput J Rafferty I Bucan M Cann HM Hardy JA Rosenberg NA Singleton AB Genotype, haplotype and copy-number variation in worldwide human populations Nature 2008 451 998 1003 18288195 10.1038/nature06742

[B45] Wang C Zollner S Rosenberg NA A Quantitative Comparison of the Similarity between Genes and Geography in Worldwide Human Populations PLoS Genet 2012 8 e1002886 22927824 10.1371/journal.pgen.1002886 PMC3426559

[B46] Marais BJ Gie RP Schaaf HS Starke JR Hesseling AC Donald PR Beyers N A proposed radiological classification of childhood intra-thoracic tuberculosis Pediatr Radiol 2004 34 886 894 15300340 10.1007/s00247-004-1238-0

[B47] Baldi P Long AD A Bayesian framework for the analysis of microarray expression data: regularized t -test and statistical inferences of gene changes Bioinformatics 2001 17 509 519 11395427 10.1093/bioinformatics/17.6.509

[B48] Storey JD Tibshirani R Statistical significance for genomewide studies Proc Natl Acad Sci USA 2003 100 9440 9445 12883005 10.1073/pnas.1530509100 PMC170937

[B49] Efron B Tibshirani RJ Improvements on cross-validation: the .632+ bootstrap method J American Statistical Association 1997 92 548 560

[B50] World Health Organization (WHO) Pneumonia Vaccine Trial Investigators Group Standardization of interpretation of chest radiographs for the diagnosis of pneumonia in children 2001 Geneva, Switzerland: WHO

