# Supplementary material for: A predictive signature gene set for discriminating active from latent tuberculosis in Warao Amerindian children
Source: BMC Genomics. 2013 Feb 1;14:74. doi: 10.1186/1471-2164-14-74 (PMC3600014; doi:10.1186/1471-2164-14-74)
Supplement: Additional file 1: Table S1 — Detailed characteristics of 27 children with TB, LTBI and HC in which microarray analyses were performed. [file 1471-2164-14-74-S1.pdf]

Table S1. Detailed characteristics of children with TB, LTBI and HC in which microarray analyses were performed.

Diagnosis	Sex	Age (years)	Culture result	TST (mm)	QFT-GIT	CXR findings
Active TB	Female	1,1	Negative	10	Indeterminate	Ghon focus with cavitation and bilateral bronchopneumonic consolidation, lymph node disease with tracheal compression
Active TB	Female	1,8	Negative	0	Positive	Ghon focus with unilateral bronchopneumonic consolidation
Active TB	Male	2,3	Negative	3	Positive	Ghon focus with bilateral bronchopneumonic consolidation, lymph node disease with bronchial compression and unilateral hyperinflation
Active TB	Female	7,5	Negative	6	Positive	Adult-type disease with unilateral cavitation and bilateral bronchopneumonic consolidation, lymph node disease with bronchial compression
Active TB	Female	8,9	Negative	0	Positive	Ghon focus with bilateral bronchopneumonic consolidation, lymph node disease with tracheal compression, pleural effusion
Active TB	Male	10,5	Negative	15	Positive	Adult-type disease with unilateral bronchopneumonic consolidation, lymph node disease with unilateral hyperinflation
Active TB	Female	11,5	Positive	18	Positive	Adult-type disease with bilateral bronchopneumonic consolidation, lymph node disease
Active TB	Female	12,5	Negative	18	Positive	Adult-type disease with unilateral cavitation, lymph node disease with lobar alveolar consolidation and unilateral hyperinflation
Active TB	Female	14,5	Positive	14	Positive	Adult-type disease with bilateral cavitation and unilateral bronchopneumonic consolidation
Latent TB infection	Male	2,2	Negative	13	Positive	No abnormalities observed
Latent TB infection	Female	3,0	Negative	11	Positive	No abnormalities observed
Latent TB infection	Male	7,0	Not performed	20	Positive	No abnormalities observed
Latent TB infection	Male	7,3	Not performed	14	Positive	No abnormalities observed
Latent TB infection	Male	9,0	Not performed	14	Positive	No abnormalities observed
Latent TB infection	Female	10,5	Not performed	10	Positive	No abnormalities observed
Latent TB infection	Female	12,7	Not performed	14	Positive	No abnormalities observed
Latent TB infection	Male	13,9	Not performed	18	Positive	No abnormalities observed
Latent TB infection	Male	14,6	Not performed	10	Positive	No abnormalities observed
Healthy control	Female	1,3	Negative	0	Negative	No abnormalities observed
Healthy control	Female	3,5	Negative	0	Negative	No abnormalities observed
Healthy control	Female	4,1	Negative	0	Negative	No abnormalities observed
Healthy control	Female	7,8	Not performed	0	Negative	No abnormalities observed
Healthy control	Male	8,0	Not performed	0	Negative	No abnormalities observed
Healthy control	Female	8,1	Not performed	0	Negative	No abnormalities observed
Healthy control	Male	9,6	Not performed	0	Negative	No abnormalities observed
Healthy control	Female	11,0	Not performed	0	Negative	No abnormalities observed
Healthy control	Female	11,5	Not performed	0	Negative	No abnormalities observed
