# Supplementary material for: A predictive signature gene set for discriminating active from latent tuberculosis in Warao Amerindian children
Source: BMC Genomics. 2013 Feb 1;14:74. doi: 10.1186/1471-2164-14-74 (PMC3600014; doi:10.1186/1471-2164-14-74)
Supplement: Additional file 2: Table S2 — Enriched gene sets in the 116 gene set. [file 1471-2164-14-74-S2.pdf]

Table S2. Enriched gene sets in the 116 gene set.						
**Category**	**Term**	**Genes**	**Count**	**Modifid Fisher exact P value (EASE Score)**	**Corrected P value (Benjamini-Hochberg)**	**Genes**
SP_PIR_KEYWORDS	synapse	5	4,9019608	2,4E-02	9,9E-01	CHRNA7, ACHE, AMPH, CHRM2, GABRA6
	neurotransmitter receptor	3	2,9411765	3,4E-02	9,7E-01	HTR1B, CHRNA7, CHRM2
	phosphoprotein	47	46,0784314	3,9E-02	9,4E-01	KLHDC7A, SFN, METTL13, SNX17, ITGA6, SNED1, SFRS7, CD8A, CD8B, TMEM40, HACL1, WDR82, ACAD11, RNF168, NCBP2, SEPT8, SOX4, ZNF192, HEY2, HTR1B, NDUFAF4, FRK, CHRM2, AMPH, PON1, WASL, VPS37A, CNTLN, GLDC, CTNNAL1, TRAF1, TUBAL3, RGS10, SART1, LAYN, HBD, CSDA, KRT8, ANAPC5, CHRNA7, SALL1, SC65, DDX5, POLRMT, RTN2, ARMCX3, DDX3Y
	cell membrane	17	16,6666667	8,9E-02	9,9E-01	S100A8, ITGA6, CD8A, CD8B, GPR78, GABRA6, HTR1B, CHRM2, CALN1, ACHE, SLC26A5, CTNNAL1, OR10A5, CLEC2A, TAS2R46, CHRNA7, KISS1R
	mrna splicing	4	3,9215686	9,1E-02	9,8E-01	DDX5, NCBP2, SFRS7, SART1
	heterodimer	3	2,9411765	9,7E-02	9,7E-01	CD8B, S100A8, ITGA6
UP_SEQ_FEATURE	calcium-binding region 1; low affinity	2	1,9607843	8,8E-02	1,0E+00	S100A8, S100P
GOTERM_BP_FAT	GO:0046620~regulation of organ growth	3	2,9411765	1,1E-02	1,0E+00	CSDA, FGF2, HEY2
	GO:0042127~regulation of cell proliferation	10	9,8039216	2,3E-02	1,0E+00	SFN, FGF2, PGGT1B, NKX2-5, SOX4, HEY2, FRK, CHRNA7, KISS1R, CDKN2D
	GO:0008361~regulation of cell size	5	4,9019608	2,4E-02	1,0E+00	ATP6V0E2, DDX5, CDKN2D, FGF2, SFN
	GO:0044057~regulation of system process	6	5,8823529	2,4E-02	1,0E+00	HTR1B, KISS1R, NKX2-5, S100P, ACHE, CHRM2
	GO:0060045~positive regulation of cardiac muscle cell proliferation	2	1,9607843	2,6E-02	9,9E-01	FGF2, HEY2
	GO:0016202~regulation of striated muscle tissue development	3	2,9411765	2,9E-02	9,9E-01	NKX2-5, FGF2, HEY2
	GO:0048634~regulation of muscle development	3	2,9411765	3,0E-02	9,8E-01	NKX2-5, FGF2, HEY2
	GO:0051050~positive regulation of transport	5	4,9019608	3,1E-02	9,7E-01	KISS1R, NKX2-5, ACHE, NCBP2, PON1
	GO:0050804~regulation of synaptic transmission	4	3,9215686	3,6E-02	9,8E-01	HTR1B, KISS1R, S100P, ACHE
	GO:0060341~regulation of cellular localization	5	4,9019608	4,3E-02	9,8E-01	HTR1B, KISS1R, ACHE, FGF2, NCBP2
	GO:0051969~regulation of transmission of nerve impulse	4	3,9215686	4,4E-02	9,8E-01	HTR1B, KISS1R, S100P, ACHE
	GO:0031644~regulation of neurological system process	4	3,9215686	4,8E-02	9,8E-01	HTR1B, KISS1R, S100P, ACHE
	GO:0032411~positive regulation of transporter activity	2	1,9607843	5,2E-02	9,8E-01	NKX2-5, PON1
	GO:0032535~regulation of cellular component size	5	4,9019608	5,6E-02	9,8E-01	ATP6V0E2, DDX5, CDKN2D, FGF2, SFN
	GO:0055024~regulation of cardiac muscle tissue development	2	1,9607843	5,7E-02	9,7E-01	FGF2, HEY2
	GO:0055021~regulation of cardiac muscle growth	2	1,9607843	5,7E-02	9,7E-01	FGF2, HEY2
	GO:0060043~regulation of cardiac muscle cell proliferation	2	1,9607843	5,7E-02	9,7E-01	FGF2, HEY2
	GO:0016477~cell migration	5	4,9019608	5,9E-02	9,7E-01	POU3F3, S100P, FGF2, ITGA6, PODXL
	GO:0051094~positive regulation of developmental process	5	4,9019608	6,1E-02	9,7E-01	CHRNA7, NKX2-5, FGF2, HEY2, SART1
	GO:0060420~regulation of heart growth	2	1,9607843	6,2E-02	9,6E-01	FGF2, HEY2
	GO:0043067~regulation of programmed cell death	9	8,8235294	6,6E-02	9,6E-01	NKX2-5, POU3F3, SOX4, TRAF1, CSDA, CDKN2D, FGF2, SART1, SFN
	GO:0010941~regulation of cell death	9	8,8235294	6,7E-02	9,6E-01	NKX2-5, POU3F3, SOX4, TRAF1, CSDA, CDKN2D, FGF2, SART1, SFN
	GO:0008284~positive regulation of cell proliferation	6	5,8823529	6,9E-02	9,6E-01	CHRNA7, NKX2-5, SOX4, FGF2, HEY2, PGGT1B
	GO:0007268~synaptic transmission	5	4,9019608	7,4E-02	9,6E-01	HTR1B, ACHE, AMOH, FGF2, GABRA6
	GO:0048870~cell motility	5	4,9019608	8,1E-02	9,7E-01	POU3F3, S100P, FGF2, ITGA6, PODXL
	GO:0051674~localization of cell	5	4,9019608	8,1E-02	9,7E-01	POU3F3, S100P, FGF2, ITGA6, PODXL
	GO:0009890~negative regulation of biosynthetic process	7	6,8627451	8,4E-02	9,7E-01	HTR1B, NKX2-5, POU3F3, CSDA, FGF2, HEY2, PGGT1B
	GO:0044093~positive regulation of molecular function	7	6,8627451	9,1E-02	9,7E-01	CHRNA7, KISS1R, NKX2-5, ANAPC5, CHRM2, FGF2, PON1
	GO:0043154~negative regulation of caspase activity	2	1,9607843	9,2E-02	9,7E-01	CDKN2D, SFN
	GO:0051046~regulation of secretion	4	3,9215686	9,3E-02	9,6E-01	HTR1B, KISS1R, ACHE, FGF2
	GO:0010605~negative regulation of macromolecule metabolic process	8	7,8431373	9,5E-02	9,6E-01	NKX2-5, POU3F3, ANAPC5, CSDA, FGF2, HEY2, PON1, PGGT1B
	GO:0031128~developmental induction	2	1,9607843	9,6E-02	9,6E-01	FGF2, SALL1
	GO:0045168~cell-cell signaling involved in cell fate specification	2	1,9607843	9,6E-02	9,6E-01	FGF2, SALL1
GOTERM_CC_FAT	GO:0043235~receptor complex	5	4,9019608	3,0E-03	4,0E-01	CD8A, CD8B, CHRNA7, GABRA6, ITGA6
	GO:0030054~cell junction	7	6,8627451	5,0E-02	9,9E-01	CHRNA7, ACHE, AMPH, CHRM2, GABRA6, ITGA6, LAYN
	GO:0043025~cell soma	4	3,9215686	5,6E-02	9,6E-01	EFHC1, S100P, CHRM2, GABRA6
	GO:0042101~T cell receptor complex	2	1,9607843	6,0E-02	9,3E-01	CD8A, CD8B
GOTERM_MF_FAT	GO:0042165~neurotransmitter binding	5	4,9019608	2,7E-03	4,9E-01	CHRNA7, KISS1R, ACHE, CHRM2, GABRA6
	GO:0008144~drug binding	4	3,9215686	4,0E-03	3,9E-01	HTR1B, CHRM2, GABRA6, PGGT1B
	GO:0042166~acetylcholine binding	3	2,9411765	6,1E-03	4,0E-01	CHRNA7, ACHE, CHRM2
	GO:0030594~neurotransmitter receptor activity	4	3,9215686	1,6E-02	6,4E-01	CHRNA7, KISS1R, CHRM2, GABRA6
	GO:0043176~amine binding	4	3,9215686	2,4E-02	7,0E-01	HTR1B, CHRNA7, ACHE, CHRM2
	GO:0048037~cofactor binding	5	4,9019608	5,1E-02	8,9E-01	HACL1, ERO1L, ACOT7, GLDC, ACAD11
	GO:0043236~laminin binding	2	1,9607843	6,0E-02	8,9E-01	ACHE, ITGA6
	GO:0001540~beta-amyloid binding	2	1,9607843	7,1E-02	9,0E-01	CHRNA7, ACHE
	GO:0050662~coenzyme binding	4	3,9215686	8,2E-02	9,0E-01	HACL1, ERO1L, ACOT7, ACAD11
	GO:0042288~MHC class I protein binding	2	1,9607843	8,6E-02	8,9E-01	CD8A, CD8B
	GO:0030291~protein serine/threonine kinase inhibitor activity	2	1,9607843	9,1E-02	8,8E-01	CDKN2D, SFN
	GO:0015464~acetylcholine receptor activity	2	1,9607843	9,7E-02	8,8E-01	CHRNA7, CHRM2
KEGG_PATHWAY	regulation of actin cytoskeleton	5	4,9019608	4,0E-02	8,1E-01	WASL, CHRM2, FGF2, ITGA6, MOS
	spliceosome	4	3,9215686	4,0E-02	5,7E-01	DDX5, NCBP2, SFRS7, SART1
	hematopoietic cell lineage	3	2,9411765	9,6E-02	7,5E-01	CD8A, CD8B, ITGA6
INTERPRO	S100/CaBP-9k-type, calcium binding	3	2,9411765	9,7E-03	8,8E-01	S100A8, S100P, CALN1
	EF-HAND2	5	4,9019608	2,8E-02	9,6E-01	EFHC1, S100A8, S100P, CALN1, CAPN12
	EF-HAND1	5	4,9019608	2,9E-02	8,8E-01	EFHC1, S100A8, S100P, CALN1, CAPN12
	EF-Hand type	5	4,9019608	3,8E-02	8,8E-01	EFHC1, S100A8, S100P, CALN1, CAPN12
PIR_SUPERFAMILY	PIRSF001321:ATP-dependent RNA helicase	2	1,9607843	8,2E-02	1,0E+00	DDX3Y, DDX5
