# Supplementary material for: A predictive signature gene set for discriminating active from latent tuberculosis in Warao Amerindian children
Source: BMC Genomics. 2013 Feb 1;14:74. doi: 10.1186/1471-2164-14-74 (PMC3600014; doi:10.1186/1471-2164-14-74)
Supplement: Additional file 3: Table S3 — Sheet 1. Minimal discriminatory gene set to discriminate between TB and LTBI. Sheet 2. Minimal discriminatory gene set to discriminate between TB, LTBI and HC. Sheet 3. Variable frequencies estimated by the bootstrapping procedure implemented in VarSelRF (1). Sheet 4. Variable frequencies estimated by the bootstrapping procedure implemented in VarSelRF (2). [file 1471-2164-14-74-S3.pdf]

Table S3 Sheet 1. Minimal discriminatory gene set to discriminate between TB and LTBI.

	ProbeID	Gene	function
Berry *et al.* [4]	ILMN_1698218	Traf1[a]	TNF receptor-associated factor 1
	ILMN_1770356	Polrmt	polymerase (RNA) mitochondrial (DNA directed)
	ILMN_1785095	Atp6v0e2	ATPase, H+ transporting V0 subunit e2
Maertzdorf *et al.* [5]	A_23_P89902	rtn2	reticulon 2
	A_24_P89891	Traf1	TNF receptor-associated factor 1
	A_32_P195291	rnf168	ring finger protein 168
Maertzdorf *et al.* [6]	A_23_P434809	S100A8	S100 calcium binding protein A8
	A_24_P89891	Traf1	TNF receptor-associated factor 1
This study	2435989	S100A8	S100 calcium binding protein A8
	3026216	CHRM2	cholinergic receptor, muscarinic 2
	3197955	Gldc	glycine dehydrogenase (decarboxylating)
	3360417	hbd	hemoglobin, delta
	combined	S100A8	S100 calcium binding protein A8
		CHRM2	cholinergic receptor, muscarinic 2
		Gldc	glycine dehydrogenase (decarboxylating)
		hbd	hemoglobin, delta
		Traf1	TNF receptor-associated factor 1
		Polrmt	polymerase (RNA) mitochondrial (DNA directed)
		Atp6v0e2	ATPase, H+ transporting V0 subunit e2
		rtn2	reticulon 2
		rnf168	ring finger protein 168

[a]Overlapping genes (genes that were selected in more than one dataset) are highlighted in yellow

Table S3 Sheet 2. Minimal discriminatory gene set to discriminate between TB, LTBI and HC.			
	ProbeID	Gene	function
Berry *et al.* [4]	ILMN_1740265	ACOT7[a]	acyl-CoA thioesterase 7
	ILMN_2334760	ARMCX3	armadillo repeat containing, X-linked 3
	ILMN_2334765	ARMCX3	armadillo repeat containing, X-linked 3
	ILMN_1785095	Atp6v0e2	ATPase, H+ transporting V0 subunit e2
	ILMN_1694798	C5orf28	chromosome 5 open reading frame 28
	ILMN_2150465	C5orf28	chromosome 5 open reading frame 28
	ILMN_1804834	C6orf130	chromosome 6 open reading frame 130
	ILMN_1706275	c8orf33	chromosome 8 open reading frame 33
	ILMN_2098325	c8orf33	chromosome 8 open reading frame 33
	ILMN_2101034	CAPN12	calpain 12
	ILMN_1787514	CAPN12	calpain 12
	ILMN_1760374	Cd8a	CD8a molecule
	ILMN_2354191	CD8B	CD8b molecule
	ILMN_1748601	CD8B	CD8b molecule
	ILMN_1772627	D4S234E	DNA segment on chromosome 4 (unique) 234 expressed sequence
	ILMN_1784977	Dohh	deoxyhypusine hydroxylase/monooxygenase
	ILMN_1798681	Gabra6	gamma-aminobutyric acid (GABA) A receptor, alpha 6
	ILMN_2289775	HAX1	HCLS1 associated protein X-1
	ILMN_2398903	HAX1	HCLS1 associated protein X-1
	ILMN_1815527	hbd	hemoglobin, delta
	ILMN_2359287	itga6	integrin, alpha 6
	ILMN_2267488	itga6	integrin, alpha 6
	ILMN_1752591	LEPROTL1	leptin receptor overlapping transcript-like 1
	ILMN_1659524	LOC100131149	NADH dehydrogenase (ubiquinone) 1 alpha subcomplex, assembly factor 4; similar to HSPC125
	ILMN_1790575	METTL13	methyltransferase like 13
	ILMN_1720442	NCBP2	nuclear cap binding protein subunit 2, 20kDa
	ILMN_1659524	Ndufaf4	NADH dehydrogenase (ubiquinone) 1 alpha subcomplex, assembly factor 4; similar to HSPC125
	ILMN_1723632	PIGC	phosphatidylinositol glycan anchor biosynthesis, class C
	ILMN_2397571	PIGC	phosphatidylinositol glycan anchor biosynthesis, class C
	ILMN_1685357	rtn2	reticulon 2
	ILMN_1680145	sart1	squamous cell carcinoma antigen recognized by T cells
	ILMN_1698218	Traf1	TNF receptor-associated factor 1
	ILMN_1711139	vps37a	vacuolar protein sorting 37 homolog A (S. cerevisiae)
	ILMN_1666004	WASL	Wiskott-Aldrich syndrome-like
	ILMN_1679655	wdr82	WD repeat domain 82
Maertzdorf *et al.* [5]	A_23_P159335	CD8B	CD8b molecule
	A_24_P89891	Traf1	TNF receptor-associated factor 1
	A_23_P87346	hbd	hemoglobin, delta
Maertzdorf *et al.* [6]	A_23_P377830	ANAPC5	anaphase promoting complex subunit 5
	A_23_P61960	Atp6v0e2	ATPase, H+ transporting V0 subunit e2
	A_23_P33119	c8orf33	chromosome 8 open reading frame 33
	A_24_P79040	CAPN12	calpain 12
	A_32_P163247	Cd8a	CD8a molecule
	A_23_P157795	CTNNAL1	catenin (cadherin-associated protein), alpha-like 1
	A_23_P121182	Hacl1	2-hydroxyacyl-CoA lyase 1
	A_23_P210176	itga6	integrin, alpha 6
	A_32_P112546	krt8	keratin 8 pseudogene 9; similar to keratin 8; keratin 8
	A_32_P112546	KRT8P9	keratin 8 pseudogene 9; similar to keratin 8; keratin 8
	A_23_P215931	LEPROTL1	leptin receptor overlapping transcript-like 1
	A_24_P171983	LOC100131149	NADH dehydrogenase (ubiquinone) 1 alpha subcomplex, assembly factor 4; similar to HSPC125
	A_32_P112546	LOC149501	keratin 8 pseudogene 9; similar to keratin 8; keratin 8
	A_24_P157424	NCBP2	nuclear cap binding protein subunit 2, 20kDa
	A_23_P155316	NCBP2	nuclear cap binding protein subunit 2, 20kDa
	A_24_P171983	Ndufaf4	NADH dehydrogenase (ubiquinone) 1 alpha subcomplex, assembly factor 4; similar to HSPC125
	A_24_P409230	OR10A2	olfactory receptor, family 10, subfamily A, member 2
	A_23_P131096	Polrmt	polymerase (RNA) mitochondrial (DNA directed)
	A_32_P195291	rnf168	ring finger protein 168
	A_24_P567454	rnf168	ring finger protein 168
	A_23_P89902	rtn2	reticulon 2
	A_23_P434809	S100A8	S100 calcium binding protein A8
	A_32_P70315	timp4	TIMP metallopeptidase inhibitor 4
	A_24_P89891	Traf1	TNF receptor-associated factor 1
	A_24_P379939	wdr82	WD repeat domain 82
This study	3026216	CHRM2	cholinergic receptor, muscarinic 2
	3197955	Gldc	glycine dehydrogenase (decarboxylating)
	2394626	ACOT7	acyl-CoA thioesterase 7
	2474479	snx17	sorting nexin 17
	3360417	hbd	hemoglobin, delta
	3444525	TAS2R46	taste receptor, type 2, member 46
Combined		ACOT7	acyl-CoA thioesterase 7
		ANAPC5	anaphase promoting complex subunit 5
		ARMCX3	armadillo repeat containing, X-linked 3
		Atp6v0e2	ATPase, H+ transporting V0 subunit e2
		C5orf28	chromosome 5 open reading frame 28
		C6orf130	chromosome 6 open reading frame 130
		c8orf33	chromosome 8 open reading frame 33
		CAPN12	calpain 12
		Cd8a	CD8a molecule
		CD8B	CD8b molecule
		CHRM2	cholinergic receptor, muscarinic 2
		CTNNAL1	catenin (cadherin-associated protein), alpha-like 1
		D4S234E	DNA segment on chromosome 4 (unique) 234 expressed sequence
		Dohh	deoxyhypusine hydroxylase/monooxygenase
		Gabra6	gamma-aminobutyric acid (GABA) A receptor, alpha 6
		Gldc	glycine dehydrogenase (decarboxylating)
		Hacl1	2-hydroxyacyl-CoA lyase 1
		HAX1	HCLS1 associated protein X-1
		hbd	hemoglobin, delta
		itga6	integrin, alpha 6
		krt8	keratin 8 pseudogene 9; similar to keratin 8; keratin 8
		KRT8P9	keratin 8 pseudogene 9; similar to keratin 8; keratin 8
		LEPROTL1	leptin receptor overlapping transcript-like 1
		LOC100131149	NADH dehydrogenase (ubiquinone) 1 alpha subcomplex, assembly factor 4; similar to HSPC125
		LOC149501	keratin 8 pseudogene 9; similar to keratin 8; keratin 8
		METTL13	methyltransferase like 13
		NCBP2	nuclear cap binding protein subunit 2, 20kDa
		Ndufaf4	NADH dehydrogenase (ubiquinone) 1 alpha subcomplex, assembly factor 4; similar to HSPC125
		OR10A2	olfactory receptor, family 10, subfamily A, member 2
		PIGC	phosphatidylinositol glycan anchor biosynthesis, class C
		Polrmt	polymerase (RNA) mitochondrial (DNA directed)
		rnf168	ring finger protein 168
		rtn2	reticulon 2
		S100A8	S100 calcium binding protein A8
		sart1	squamous cell carcinoma antigen recognized by T cells
		snx17	sorting nexin 17
		TAS2R46	taste receptor, type 2, member 46
		timp4	TIMP metallopeptidase inhibitor 4
		Traf1	TNF receptor-associated factor 1
		vps37a	vacuolar protein sorting 37 homolog A (S. cerevisiae)
		WASL	Wiskott-Aldrich syndrome-like
		wdr82	WD repeat domain 82

[a]Overlapping genes (genes that were selected in more than one dataset) are highlighted in yellow

Table S3 Sheet 3. Variable frequencies estimated by the bootstrapping procedure implemented in VarSelfRF (1). The ten genes that were selected for the optimal gene set are highlighted in yellow.

This study active latent healthy

Probe	Gene	Function	Variable frequency
3197955	Gldc	glycine dehydrogenase (decarboxylating)	0.34
3026216	CHRM2	cholinergic receptor. muscarinic 2	0.29
3444525	TAS2R46	taste receptor. type 2. member 46	0.255
2474479	snx17	sorting nexin 17	0.245
3360417	hbd	hemoglobin. delta	0.235
2394626	ACOT7	acyl-CoA thioesterase 7	0.205
2717078	S100P	S100 calcium binding protein P	0.16
3163728	Cntln	centlein. centrosomal protein	0.16
2721633	SOD3	superoxide dismutase 3. extracellular	0.15
3821079	LOC55908	hepatocellular carcinoma-associated gene TD26	0.14
2871685	Pggt1b	protein geranylgeranyltransferase type I. beta subunit	0.125
3984945	ARMCX3	armadillo repeat containing. X-linked 3	0.125
2663295	TMEM40	transmembrane protein 40	0.12
2716467	D4S234E	DNA segment on chromosome 4 (unique) 234 expressed sequence	0.11
4031692	PRY	PTPN13-like. Y-linked pseudogene 4; PTPN13-like. Y-linked pseudogene 3; PTPN13-like. Y-linked; PTPN13-like. Y-linked 2	0.1
2323285	Klhdc7a	kelch domain containing 7A	0.095
2326774	SFN	stratifin	0.095
3061942	PON1	paraoxonase 1	0.095
3474984	ANAPC5	anaphase promoting complex subunit 5	0.095
3309602	Rgs10	regulator of G-protein signaling 10	0.09
3691326	sall1	sal-like 1 (Drosophila)	0.09
3851250	ZNF20	zinc finger protein 20	0.09
2695648	NPHP3	nephronophthisis 3 (adolescent); acyl-Coenzyme A dehydrogenase family. member 11	0.085
2794679	SPATA4	spermatogenesis associated 4	0.085
3392934			0.085
3757376	Sc65	synaptonemal complex protein SC65	0.085
3846214	Dohh	deoxyhypusine hydroxylase/monooxygenase	0.085
2444117	PIGC	phosphatidylinositol glycan anchor biosynthesis. class C	0.08
2742109	FGF2	fibroblast growth factor 2 (basic)	0.08
3725896			0.08
3743937			0.08
2717846	gpr78	G protein-coupled receptor 78	0.075
2838399	Gabra6	gamma-aminobutyric acid (GABA) A receptor. alpha 6	0.075
3046739	ampH	amphiphysin	0.075
2712906	rnf168	ring finger protein 168	0.07
2841382			0.07
2897899	Sox4	SRY (sex determining region Y)-box 4	0.065
2970897	FRK	fyn-related kinase	0.065
3057550	STYXL1	serine/threonine/tyrosine interacting-like 1	0.065
3306585			0.065
3335802	sart1	squamous cell carcinoma antigen recognized by T cells	0.065
2497812	POU3F3	POU class 3 homeobox 3	0.06
3443913	CLEC2A	C-type lectin domain family 2. member A	0.06
3455516	KRT8P9	keratin 8 pseudogene 9; similar to keratin 8; keratin 8	0.06
3849022	ZNF414	zinc finger protein 414	0.06
3223738	Traf1	TNF receptor-associated factor 1	0.055
2562965	CD8B	CD8b molecule	0.05
2808612	Mrps30	mitochondrial ribosomal protein S30	0.05
2965674	LOC100131149	NADH dehydrogenase (ubiquinone) 1 alpha subcomplex. assembly factor 4; similar to HSPC125	0.05
3318961	OR10A2	olfactory receptor. family 10. subfamily A. member 2	0.05
2536071	SNED1	sushi. nidogen and EGF-like domains 1	0.045
2556017	C2orf86	chromosome 2 open reading frame 86	0.045
2968399			0.045
3865422	rtn2	reticulon 2	0.045
3867326	MAMSTR	MEF2 activating motif and SAP domain containing transcriptional regulator	0.045
2562932	Cd8a	CD8a molecule	0.04
2887449	NKX2-5	NK2 transcription factor related. locus 5 (Drosophila)	0.04
3073013	PODXL	podocalyxin-like	0.04
3136167	mos	v-mos Moloney murine sarcoma viral oncogene homolog	0.04
3219621	CTNNAL1	catenin (cadherin-associated protein). alpha-like 1	0.04
3850445	CDKN2D	cyclin-dependent kinase inhibitor 2D (p19. inhibits CDK4)	0.04
2544565	DNAJC27	DnaJ (Hsp40) homolog. subfamily C. member 27	0.035
2616204	Fbxl2	F-box and leucine-rich repeat protein 2	0.035
2855578	C5orf28	chromosome 5 open reading frame 28	0.035
2875454	39692	septin 8	0.035
3031181	Atp6v0e2	ATPase. H+ transporting V0 subunit e2	0.035
3551485	EML1	echinoderm microtubule associated protein like 1	0.035
2435989	S100A8	S100 calcium binding protein A8	0.03
2536436			0.03
2591942	ORMDL1	ORM1-like 1 (S. cerevisiae)	0.03
2674414			0.03
3055466	CALN1	calneuron 1	0.03
3064361	ache	acetylcholinesterase (Yt blood group)	0.03
3092276	LEPROTL1	leptin receptor overlapping transcript-like 1	0.03
3121023	c8orf33	chromosome 8 open reading frame 33	0.03
3335774	sart1	squamous cell carcinoma antigen recognized by T cells	0.03
3348568	LAYN	layilin	0.03
3444252	CSDAP1	cold shock domain protein A; cold shock domain protein A pseudogene 1	0.03
3474963			0.03
3851441	ZNF442	zinc finger protein 442	0.03
3917431			0.03
2328565			0.025
2676041	wdr82	WD repeat domain 82	0.025
2713074	NCBP2	nuclear cap binding protein subunit 2. 20kDa	0.025
2900299	ZNF192	zinc finger protein 192	0.025
2901043			0.025
3065684	SLC26A5	solute carrier family 26. member 5 (prestin)	0.025
2664395	Hacl1	2-hydroxyacyl-CoA lyase 1	0.02
2961647	HTR1B	5-hydroxytryptamine (serotonin) receptor 1B	0.02
3335830			0.02
3749672			0.02
3819016	LOC100131801	similar to hCG2036585	0.02
3836057	LRRC68	leucine rich repeat containing 68	0.02
3844656	Polrmt	polymerase (RNA) mitochondrial (DNA directed)	0.02
3861503	CAPN12	calpain 12	0.02
2367287	METTL13	methyltransferase like 13	0.015
2515627	itga6	integrin. alpha 6	0.015
2548970	sfrs7	splicing factor. arginine/serine-rich 7. 35kDa	0.015
2924492	HEY2	hypothetical LOC100129733; hairy/enhancer-of-split related with YRPW motif 2	0.015
2953435	C6orf130	chromosome 6 open reading frame 130	0.015
4030162	DDX3Y	DEAD (Asp-Glu-Ala-Asp) box polypeptide 3. Y-linked	0.015
2360158	HAX1	HCLS1 associated protein X-1	0.01
2454935	ANGEL2	angel homolog 2 (Drosophila)	0.01
3070712	WASL	Wiskott-Aldrich syndrome-like	0.01
3274898	TUBAL3	tubulin. alpha-like 3	0.01
3766920	DDX5	DEAD (Asp-Glu-Ala-Asp) box polypeptide 5	0.01
2663130	timp4	TIMP metallopeptidase inhibitor 4	0.005
2910236	EFHC1	EF-hand domain (C-terminal) containing 1	0.005
2994835	Chn2	chimerin (chimaerin) 2	0.005
3087555	vps37a	vacuolar protein sorting 37 homolog A (S. cerevisiae)	0.005
3375612	RAB3IL1	RAB3A interacting protein (rabin3)-like 1	0.005
3564790	ERO1L	ERO1-like (S. cerevisiae)	0.005
3587226	CHRNA7	CHRNA7 (cholinergic receptor. nicotinic. alpha 7. exons 5-10) and FAM7A (family with sequence similarity 7A. exons A-E) f u	0.005
3815268	Kiss1r	KISS1 receptor	0.005

Berry *et al.*[4] active latent healthy otherdisease

Probe	Gene	Function	Variable frequency
ILMN_2397571	PIGC	phosphatidylinositol glycan anchor biosynthesis. class C	1
ILMN_1723632	PIGC	phosphatidylinositol glycan anchor biosynthesis. class C	0.985
ILMN_2334760	ARMCX3	armadillo repeat containing. X-linked 3	0.95
ILMN_1785095	Atp6v0e2	ATPase. H+ transporting V0 subunit e2	0.94
ILMN_2398903	HAX1	HCLS1 associated protein X-1	0.905
ILMN_1711139	vps37a	vacuolar protein sorting 37 homolog A (S. cerevisiae)	0.86
ILMN_1790575	METTL13	methyltransferase like 13	0.815
ILMN_1744963	ERO1L	ERO1-like (S. cerevisiae)	0.725
ILMN_1685357	rtn2	reticulon 2	0.705
ILMN_1752591	LEPROTL1	leptin receptor overlapping transcript-like 1	0.695
ILMN_1706275	c8orf33	chromosome 8 open reading frame 33	0.65
ILMN_1679655	wdr82	WD repeat domain 82	0.64
ILMN_1750658	HAX1	HCLS1 associated protein X-1	0.625
ILMN_1748883	CDKN2D	cyclin-dependent kinase inhibitor 2D (p19. inhibits CDK4)	0.6
ILMN_1801216	S100P	S100 calcium binding protein P	0.595
ILMN_1698406	ORMDL1	ORM1-like 1 (S. cerevisiae)	0.51
ILMN_1694798	C5orf28	chromosome 5 open reading frame 28	0.495
ILMN_1666004	WASL	Wiskott-Aldrich syndrome-like	0.49
ILMN_1815745	Sox4	SRY (sex determining region Y)-box 4	0.485
ILMN_2334765	ARMCX3	armadillo repeat containing. X-linked 3	0.435
ILMN_2161832	vps37a	vacuolar protein sorting 37 homolog A (S. cerevisiae)	0.42
ILMN_2136446	CTNNAL1	catenin (cadherin-associated protein). alpha-like 1	0.405
ILMN_1778121	CALN1	calneuron 1	0.365
ILMN_1721901	CTNNAL1	catenin (cadherin-associated protein). alpha-like 1	0.31
ILMN_1732810	snx17	sorting nexin 17	0.29
ILMN_1659524	LOC100131149	NADH dehydrogenase (ubiquinone) 1 alpha subcomplex. assembly factor 4; similar to HSPC125	0.255
ILMN_1726743	Mrps30	mitochondrial ribosomal protein S30	0.255
ILMN_1669005	CD8B	CD8b molecule	0.225
ILMN_1772627	D4S234E	DNA segment on chromosome 4 (unique) 234 expressed sequence	0.19
ILMN_1778836	sfrs7	splicing factor. arginine/serine-rich 7. 35kDa	0.18
ILMN_1685834	ampH	amphiphysin	0.175
ILMN_2353732	Cd8a	CD8a molecule	0.175
ILMN_1804834	C6orf130	chromosome 6 open reading frame 130	0.17
ILMN_1720442	NCBP2	nuclear cap binding protein subunit 2. 20kDa	0.145
ILMN_2359287	itga6	integrin. alpha 6	0.145
ILMN_2210729	STYXL1	serine/threonine/tyrosine interacting-like 1	0.135
ILMN_1760374	Cd8a	CD8a molecule	0.12
ILMN_1740265	ACOT7	acyl-CoA thioesterase 7	0.11
ILMN_1764714	LOC55908	hepatocellular carcinoma-associated gene TD26	0.105
ILMN_1787514	CAPN12	calpain 12	0.1
ILMN_2373377	rtn2	reticulon 2	0.1
ILMN_1748601	CD8B	CD8b molecule	0.08
ILMN_1736340	ANGEL2	angel homolog 2 (Drosophila)	0.075
ILMN_1815527	hbd	hemoglobin. delta	0.075
ILMN_2098325	c8orf33	chromosome 8 open reading frame 33	0.075
ILMN_1798681	Gabra6	gamma-aminobutyric acid (GABA) A receptor. alpha 6	0.07
ILMN_1663754	ZNF442	zinc finger protein 442	0.05
ILMN_1670377	ZNF20	zinc finger protein 20	0.05
ILMN_1729801	S100A8	S100 calcium binding protein A8	0.05
ILMN_1749115	rtn2	reticulon 2	0.05
ILMN_1805344	DDX5	DEAD (Asp-Glu-Ala-Asp) box polypeptide 5	0.045
ILMN_2354191	CD8B	CD8b molecule	0.04
ILMN_1722407			0.035
ILMN_1757958	SLC26A5	solute carrier family 26. member 5 (prestin)	0.03
ILMN_1768482	Cd8a	CD8a molecule	0.03
ILMN_2267488	itga6	integrin. alpha 6	0.025
ILMN_1698218	Traf1	TNF receptor-associated factor 1	0.02
ILMN_1810436	DNAJC27	DnaJ (Hsp40) homolog. subfamily C. member 27	0.02
ILMN_2101034	CAPN12	calpain 12	0.02
ILMN_2150465	C5orf28	chromosome 5 open reading frame 28	0.02
ILMN_2156786	Pggt1b	protein geranylgeranyltransferase type I. beta subunit	0.02
ILMN_2222786	ZNF192	zinc finger protein 192	0.02
ILMN_2332250	ACOT7	acyl-CoA thioesterase 7	0.02
ILMN_1762061			0.015
ILMN_2045975	ORMDL1	ORM1-like 1 (S. cerevisiae)	0.015
ILMN_2297511	PODXL	podocalyxin-like	0.015
ILMN_2333219	ache	acetylcholinesterase (Yt blood group)	0.015
ILMN_1680145	sart1	squamous cell carcinoma antigen recognized by T cells	0.01
ILMN_1733538	Rgs10	regulator of G-protein signaling 10	0.01
ILMN_1749034	CALN1	calneuron 1	0.01
ILMN_1770356	Polrmt	polymerase (RNA) mitochondrial (DNA directed)	0.01
ILMN_1806754	Gldc	glycine dehydrogenase (decarboxylating)	0.01
ILMN_2360929	C2orf86	chromosome 2 open reading frame 86	0.01
ILMN_2413158	PODXL	podocalyxin-like	0.01
ILMN_1682034	HEY2	hypothetical LOC100129733; hairy/enhancer-of-split related with YRPW motif 2	0.005
ILMN_1761058	NPHP3	nephronophthisis 3 (adolescent); acyl-Coenzyme A dehydrogenase family. member 11	0.005
ILMN_1761574	CHRM2	cholinergic receptor. muscarinic 2	0.005
ILMN_1761693	CALN1	calneuron 1	0.005
ILMN_1782788	CSDAP1	cold shock domain protein A; cold shock domain protein A pseudogene 1	0.005
ILMN_2252621	ACOT7	acyl-CoA thioesterase 7	0.005
ILMN_2297161	NCBP2	nuclear cap binding protein subunit 2. 20kDa	0.005
ILMN_2343624	METTL13	methyltransferase like 13	0.005
ILMN_2358718	CHRM2	cholinergic receptor. muscarinic 2	0.005

Maertzdorf *et al.* [5] active latent healthy

Probe	Gene	Function	Variable frequency
A_23_P87346_24390	hbd	hemoglobin. delta	0.75
A_23_P159335_43831	CD8B	CD8b molecule	0.595
A_24_P89891_13200	Traf1	TNF receptor-associated factor 1	0.565
A_23_P434809_28674	S100A8	S100 calcium binding protein A8	0.5
A_23_P131096_21786	Polrmt	polymerase (RNA) mitochondrial (DNA directed)	0.325
A_23_P371107_37642	D4S234E	DNA segment on chromosome 4 (unique) 234 expressed sequence	0.31
A_23_P157795_14744	CTNNAL1	catenin (cadherin-associated protein). alpha-like 1	0.27
A_32_P85978_26401	ZNF414	zinc finger protein 414	0.19
A_23_P357881_14587	CD8B	CD8b molecule	0.18
A_32_P95082_20313	Cntln	centlein. centrosomal protein	0.18
A_23_P147698_17912	CALN1	calneuron 1	0.175
A_23_P61960_33699	Atp6v0e2	ATPase. H+ transporting V0 subunit e2	0.155
A_24_P625382_34396	CSDAP1	cold shock domain protein A; cold shock domain protein A pseudogene 1	0.155
A_23_P89902_31771	rtn2	reticulon 2	0.145
A_23_P25224_8151	CSDAP1	cold shock domain protein A; cold shock domain protein A pseudogene 1	0.135
A_32_P163247_1481	Cd8a	CD8a molecule	0.135
A_23_P340922_3417	ZNF414	zinc finger protein 414	0.11
A_23_P218918_37607	FGF2	fibroblast growth factor 2 (basic)	0.085
A_23_P29551_17955	TMEM40	transmembrane protein 40	0.085
A_32_P98847_5271	Polrmt	polymerase (RNA) mitochondrial (DNA directed)	0.08
A_23_P106145_8032	ERO1L	ERO1-like (S. cerevisiae)	0.06
A_23_P156824_41124	HTR1B	5-hydroxytryptamine (serotonin) receptor 1B	0.06
A_23_P157963_9767	Cntln	centlein. centrosomal protein	0.06
A_23_P155316_1999	NCBP2	nuclear cap binding protein subunit 2. 20kDa	0.045
A_24_P171983_7609	LOC100131149	NADH dehydrogenase (ubiquinone) 1 alpha subcomplex. assembly factor 4; similar to HSPC125	0.045
A_24_P407866_12175	Sc65	synaptonemal complex protein SC65	0.04
A_23_P121182_35512	Hacl1	2-hydroxyacyl-CoA lyase 1	0.035
A_24_P379939_35134	wdr82	WD repeat domain 82	0.035
A_23_P109877_8855	wdr82	WD repeat domain 82	0.03
A_23_P256632_21714	Pggt1b	protein geranylgeranyltransferase type I. beta subunit	0.03
A_23_P58266_12550	S100P	S100 calcium binding protein P	0.03
A_23_P86252_29856	PIGC	phosphatidylinositol glycan anchor biosynthesis. class C	0.03
A_24_P924217_30997	Cntln	centlein. centrosomal protein	0.03
A_32_P112546_2452	KRT8P9	keratin 8 pseudogene 9; similar to keratin 8; keratin 8	0.03
A_24_P130936_10858	DDX3Y	DEAD (Asp-Glu-Ala-Asp) box polypeptide 3. Y-linked	0.025
A_24_P409071_11590	SNED1	sushi. nidogen and EGF-like domains 1	0.025
A_23_P205746_23871	EML1	echinoderm microtubule associated protein like 1	0.02
A_23_P33119_23817	c8orf33	chromosome 8 open reading frame 33	0.02
A_23_P422131_7590	Klhdc7a	kelch domain containing 7A	0.02
A_23_P63254_22500	SFN	stratifin	0.02
A_23_P101303_6134	LRRC68	leucine rich repeat containing 68	0.015
A_23_P111635_34914	STYXL1	serine/threonine/tyrosine interacting-like 1	0.015
A_23_P201672_9946	METTL13	methyltransferase like 13	0.015
A_23_P204448_15337	ANAPC5	anaphase promoting complex subunit 5	0.015
A_23_P218918_26293	FGF2	fibroblast growth factor 2 (basic)	0.015
A_24_P60845_9922	ache	acetylcholinesterase (Yt blood group)	0.015
A_32_P13795_12503	DNAJC27	DnaJ (Hsp40) homolog. subfamily C. member 27	0.015
A_23_P101476_40385	ZNF442	zinc finger protein 442	0.01
A_23_P134609_13009	WASL	Wiskott-Aldrich syndrome-like	0.01
A_23_P212497_39943	NPHP3	nephronophthisis 3 (adolescent); acyl-Coenzyme A dehydrogenase family. member 11	0.01
A_23_P214529_27293	ZNF192	zinc finger protein 192	0.01
A_23_P217797_27895	DDX3Y	DEAD (Asp-Glu-Ala-Asp) box polypeptide 3. Y-linked	0.01
A_23_P254741_40689	SOD3	superoxide dismutase 3. extracellular	0.01
A_23_P415827_24768	39692	septin 8	0.01
A_23_P58251_14827	Cpz	carboxypeptidase Z	0.01
A_23_P69652_5111	gpr78	G protein-coupled receptor 78	0.01
A_23_P74852_20384	PIGC	phosphatidylinositol glycan anchor biosynthesis. class C	0.01
A_23_P90565_6262	C2orf86	chromosome 2 open reading frame 86	0.01
A_24_P170887_28054	c8orf33	chromosome 8 open reading frame 33	0.01
A_24_P205589_15256	ACOT7	acyl-CoA thioesterase 7	0.01
A_24_P337846_33117	PON1	paraoxonase 1	0.01
A_24_P407311_7747	ERO1L	ERO1-like (S. cerevisiae)	0.01
A_24_P489695_38510	FLJ46111	keratin 8 pseudogene	0.01
A_24_P79040_41825	CAPN12	calpain 12	0.01
A_24_P911676_14409	Sox4	SRY (sex determining region Y)-box 4	0.01
A_32_P133999_16752	CALN1	calneuron 1	0.01
A_23_P14072_30261	KRT8P9	keratin 8 pseudogene 9; similar to keratin 8; keratin 8	0.005
A_23_P157352_20344	mRpS33	mitochondrial ribosomal protein S33	0.005
A_23_P16242_32942	ZNF20	zinc finger protein 20	0.005
A_23_P17275_27154	DNAJC27	DnaJ (Hsp40) homolog. subfamily C. member 27	0.005
A_23_P17955_21452	Fbxl2	F-box and leucine-rich repeat protein 2	0.005
A_23_P201676_5277	METTL13	methyltransferase like 13	0.005
A_23_P214291_26456	EFHC1	EF-hand domain (C-terminal) containing 1	0.005
A_23_P215060_3965	PODXL	podocalyxin-like	0.005
A_23_P215931_40301	LEPROTL1	leptin receptor overlapping transcript-like 1	0.005
A_23_P218918_24707	FGF2	fibroblast growth factor 2 (basic)	0.005
A_23_P252362_31993	Mrps30	mitochondrial ribosomal protein S30	0.005
A_23_P39704_18707	sfrs7	splicing factor. arginine/serine-rich 7. 35kDa	0.005
A_23_P413043_38666	Bach1	BTB and CNC homology 1. basic leucine zipper transcription factor 1	0.005
A_23_P41847_39251	Gabra6	gamma-aminobutyric acid (GABA) A receptor. alpha 6	0.005
A_23_P75800_43724	RAB3IL1	RAB3A interacting protein (rabin3)-like 1	0.005
A_23_P95851_20621	TUBAL3	tubulin. alpha-like 3	0.005
A_24_P218048_25074	ampH	amphiphysin	0.005
A_24_P230767_9868	STYXL1	serine/threonine/tyrosine interacting-like 1	0.005
A_24_P23411_10177	ARMCX3	armadillo repeat containing. X-linked 3	0.005
A_24_P277875_40830	Chn2	chimerin (chimaerin) 2	0.005
A_24_P363408_38310	HEY2	hypothetical LOC100129733; hairy/enhancer-of-split related with YRPW motif 2	0.005
A_24_P46659_26988	LOC100131149	NADH dehydrogenase (ubiquinone) 1 alpha subcomplex. assembly factor 4; similar to HSPC125	0.005
A_32_P35969_30896	CHRNA7	CHRNA7 (cholinergic receptor. nicotinic. alpha 7. exons 5-10) and FAM7A (family with sequence similarity 7A. exons A-E) fusion; cholinergic receptor. nicotinic. alpha 7	0.005

Maertzdorf *et al.* [6] active latent healthy

Probe	Gene	Function	Variable frequency
A_23_P89902	rtn2	reticulon 2	0.905
A_32_P195291	rnf168	ring finger protein 168	0.78
A_24_P89891	Traf1	TNF receptor-associated factor 1	0.495
A_24_P79040	CAPN12	calpain 12	0.49
A_23_P131096	Polrmt	polymerase (RNA) mitochondrial (DNA directed)	0.47
A_23_P157795	CTNNAL1	catenin (cadherin-associated protein). alpha-like 1	0.465
A_23_P215931	LEPROTL1	leptin receptor overlapping transcript-like 1	0.46
A_32_P163247	Cd8a	CD8a molecule	0.395
A_24_P171983	LOC100131149	NADH dehydrogenase (ubiquinone) 1 alpha subcomplex. assembly factor 4; similar to HSPC125	0.29
A_24_P409230	OR10A2	olfactory receptor. family 10. subfamily A. member 2	0.255
A_23_P377830	ANAPC5	anaphase promoting complex subunit 5	0.2
A_24_P379939	wdr82	WD repeat domain 82	0.2
A_32_P112546	KRT8P9	keratin 8 pseudogene 9; similar to keratin 8; keratin 8	0.2
A_24_P567454	rnf168	ring finger protein 168	0.185
A_24_P157424	NCBP2	nuclear cap binding protein subunit 2. 20kDa	0.17
A_23_P155316	NCBP2	nuclear cap binding protein subunit 2. 20kDa	0.165
A_23_P121182	Hacl1	2-hydroxyacyl-CoA lyase 1	0.155
A_23_P210176	itga6	integrin. alpha 6	0.115
A_23_P121875	C5orf28	chromosome 5 open reading frame 28	0.1
A_23_P33119	c8orf33	chromosome 8 open reading frame 33	0.095
A_23_P61960	Atp6v0e2	ATPase. H+ transporting V0 subunit e2	0.09
A_23_P87346	hbd	hemoglobin. delta	0.09
A_32_P70315	timp4	TIMP metallopeptidase inhibitor 4	0.08
A_24_P205589	ACOT7	acyl-CoA thioesterase 7	0.075
A_23_P159335	CD8B	CD8b molecule	0.07
A_24_P222911	sfrs7	splicing factor. arginine/serine-rich 7. 35kDa	0.065
A_24_P46659	LOC100131149	NADH dehydrogenase (ubiquinone) 1 alpha subcomplex. assembly factor 4; similar to HSPC125	0.065
A_24_P625382	CSDAP1	cold shock domain protein A; cold shock domain protein A pseudogene 1	0.06
A_23_P434809	S100A8	S100 calcium binding protein A8	0.055
A_23_P357881	CD8B	CD8b molecule	0.05
A_23_P39704	sfrs7	splicing factor. arginine/serine-rich 7. 35kDa	0.05
A_23_P41847	Gabra6	gamma-aminobutyric acid (GABA) A receptor. alpha 6	0.045
A_23_P371107	D4S234E	DNA segment on chromosome 4 (unique) 234 expressed sequence	0.04
A_32_P95082	Cntln	centlein. centrosomal protein	0.04
A_23_P28238	snx17	sorting nexin 17	0.035
A_23_P101761	Kiss1r	KISS1 receptor	0.03
A_23_P25224	CSDAP1	cold shock domain protein A; cold shock domain protein A pseudogene 1	0.03
A_23_P89941	CDKN2D	cyclin-dependent kinase inhibitor 2D (p19. inhibits CDK4)	0.03
A_23_P120194	ORMDL1	ORM1-like 1 (S. cerevisiae)	0.025
A_23_P218918	FGF2	fibroblast growth factor 2 (basic)	0.02
A_23_P95851	TUBAL3	tubulin. alpha-like 3	0.02
A_32_P387905	ERO1L	ERO1-like (S. cerevisiae)	0.02
A_23_P109877	wdr82	WD repeat domain 82	0.015
A_23_P17275	DNAJC27	DnaJ (Hsp40) homolog. subfamily C. member 27	0.015
A_23_P28233	snx17	sorting nexin 17	0.015
A_23_P340922	ZNF414	zinc finger protein 414	0.015
A_24_P170887	c8orf33	chromosome 8 open reading frame 33	0.015
A_24_P28619	ANGEL2	angel homolog 2 (Drosophila)	0.015
A_23_P130677	LOC55908	hepatocellular carcinoma-associated gene TD26	0.01
A_23_P133665	FRK	fyn-related kinase	0.01
A_23_P204448	ANAPC5	anaphase promoting complex subunit 5	0.01
A_23_P31273	ampH	amphiphysin	0.01
A_23_P86252	PIGC	phosphatidylinositol glycan anchor biosynthesis. class C	0.01
A_24_P230767	STYXL1	serine/threonine/tyrosine interacting-like 1	0.01
A_24_P28622	ANGEL2	angel homolog 2 (Drosophila)	0.01
A_24_P34575	POU3F3	POU class 3 homeobox 3	0.01
A_24_P388536	wdr82	WD repeat domain 82	0.01
A_24_P67395	KRT8P9	keratin 8 pseudogene 9; similar to keratin 8; keratin 8	0.01
A_23_P101308	LRRC68	leucine rich repeat containing 68	0.005
A_23_P106145	ERO1L	ERO1-like (S. cerevisiae)	0.005
A_23_P111635	STYXL1	serine/threonine/tyrosine interacting-like 1	0.005
A_23_P11408	PRY	PTPN13-like. Y-linked pseudogene 4; PTPN13-like. Y-linked pseudogene 3; PTPN13-like. Y-linked; PTPN13-like. Y-linked 2	0.005
A_23_P115223	HAX1	HCLS1 associated protein X-1	0.005
A_23_P125408	Dohh	deoxyhypusine hydroxylase/monooxygenase	0.005
A_23_P134609	WASL	Wiskott-Aldrich syndrome-like	0.005
A_23_P15511	DDX5	DEAD (Asp-Glu-Ala-Asp) box polypeptide 5	0.005
A_23_P157513	mos	v-mos Moloney murine sarcoma viral oncogene homolog	0.005
A_23_P16409	CAPN12	calpain 12	0.005
A_23_P205746	EML1	echinoderm microtubule associated protein like 1	0.005
A_23_P214291	EFHC1	EF-hand domain (C-terminal) containing 1	0.005
A_23_P217611	ARMCX3	armadillo repeat containing. X-linked 3	0.005
A_23_P254741	SOD3	superoxide dismutase 3. extracellular	0.005
A_23_P29551	TMEM40	transmembrane protein 40	0.005
A_23_P413043	Bach1	BTB and CNC homology 1. basic leucine zipper transcription factor 1	0.005
A_23_P69652	gpr78	G protein-coupled receptor 78	0.005
A_23_P90565	C2orf86	chromosome 2 open reading frame 86	0.005
A_24_P371053	ORMDL1	ORM1-like 1 (S. cerevisiae)	0.005
A_24_P409071	SNED1	sushi. nidogen and EGF-like domains 1	0.005
A_24_P60845	ache	acetylcholinesterase (Yt blood group)	0.005
A_24_P88031	c8orf33	chromosome 8 open reading frame 33	0.005
A_24_P911676	Sox4	SRY (sex determining region Y)-box 4	0.005
A_24_P924217	Cntln	centlein. centrosomal protein	0.005
A_32_P13795	DNAJC27	DnaJ (Hsp40) homolog. subfamily C. member 27	0.005

Table S3 Sheet 4. Variable frequencies estimated by the bootstrapping procedure implemented in VarSelfRF (2). The ten genes that were selected for the optimal gene set are highlighted in yellow.

Gene name	Variable frequencies			
	This study	Berry *et al.* [4]	Maertzdorf *et al.* [5]	Maertzdorf *et al.* [6]
hbd	0.235	0.075	0.75	0.09
S100P	0.16	0.595	0.03	0
snx17	0.245	0.29	0	0.035
ACOT7	0.205	0.11	0.01	0.075
Gldc	0.34	0.01	0	0
CHRM2	0.29	0.005	0	0
TAS2R46	0.255	0	0	0
D4S234E	0.11	0.19	0.31	0.04
CD8B	0.05	0.225	0.595	0.07
CTNNAL1	0.04	0.405	0.27	0.465
CALN1	0.03	0.365	0.175	0
PIGC	0.08	1	0.03	0.01
ampH	0.075	0.175	0.005	0.01
STYXL1	0.065	0.135	0.015	0.01
ARMCX3	0.125	0.95	0.005	0.005
LOC100131149	0.05	0.255	0.045	0.29
C5orf28	0.035	0.495	0	0.1
Atp6v0e2	0.035	0.94	0.155	0.09
LEPROTL1	0.03	0.695	0.005	0.46
c8orf33	0.03	0.65	0.02	0.095
wdr82	0.025	0.64	0.035	0.2
METTL13	0.015	0.815	0.015	0
HAX1	0.01	0.905	0	0.005
WASL	0.01	0.49	0.01	0.005
Cntln	0.16	0	0.18	0.04
LOC55908	0.14	0.105	0	0.01
Sox4	0.065	0.485	0.01	0.005
ZNF414	0.06	0	0.19	0.015
Traf1	0.055	0.02	0.565	0.495
Mrps30	0.05	0.255	0.005	0
rtn2	0.045	0.705	0.145	0.905
CDKN2D	0.04	0.6	0	0.03
Cd8a	0.04	0.175	0.135	0.395
ORMDL1	0.03	0.51	0	0.025
S100A8	0.03	0.05	0.5	0.055
NCBP2	0.025	0.145	0.045	0.17
CAPN12	0.02	0.1	0.01	0.49
Polrmt	0.02	0.01	0.325	0.47
itga6	0.015	0.145	0	0.115
C6orf130	0.015	0.17	0	0
sfrs7	0.015	0.18	0.005	0.065
SOD3	0.15	0	0.01	0.005
Pggt1b	0.125	0.02	0.03	0
TMEM40	0.12	0	0.085	0.005
PRY	0.1	0	0	0.005
PON1	0.095	0	0.01	0
ANAPC5	0.095	0	0.015	0.2
SFN	0.095	0	0.02	0
Klhdc7a	0.095	0	0.02	0
sall1	0.09	0	0	0
Rgs10	0.09	0.01	0	0
ZNF20	0.09	0.05	0.005	0
Dohh	0.085	0	0	0.005
SPATA4	0.085	0	0	0
NPHP3	0.085	0.005	0.01	0
Sc65	0.085	0	0.04	0
FGF2	0.08	0	0.085	0.02
Gabra6	0.075	0.07	0.005	0.045
gpr78	0.075	0	0.01	0.005
rnf168	0.07	0	0	0.78
FRK	0.065	0	0	0.01
sart1	0.065	0.01	0	0
POU3F3	0.06	0	0	0.01
CLEC2A	0.06	0	0	0
KRT8P9	0.06	0	0.03	0.2
OR10A2	0.05	0	0	0.255
MAMSTR	0.045	0	0	0
C2orf86	0.045	0.01	0.01	0.005
SNED1	0.045	0	0.025	0.005
mos	0.04	0	0	0.005
NKX2-5	0.04	0	0	0
PODXL	0.04	0.015	0.005	0
Fbxl2	0.035	0	0.005	0
39692	0.035	0	0.01	0
DNAJC27	0.035	0.02	0.015	0.015
EML1	0.035	0	0.02	0.005
LAYN	0.03	0	0	0
ZNF442	0.03	0.05	0.01	0
ache	0.03	0.015	0.015	0.005
CSDAP1	0.03	0.005	0.155	0.06
SLC26A5	0.025	0.03	0	0
ZNF192	0.025	0.02	0.01	0
LOC100131801	0.02	0	0	0
LRRC68	0.02	0	0.015	0.005
Hacl1	0.02	0	0.035	0.155
HTR1B	0.02	0	0.06	0
HEY2	0.015	0.005	0.005	0
DDX3Y	0.015	0	0.025	0
DDX5	0.01	0.045	0	0.005
ANGEL2	0.01	0.075	0	0.015
TUBAL3	0.01	0	0.005	0.02
vps37a	0.005	0.86	0	0
ERO1L	0.005	0.725	0.06	0.02
timp4	0.005	0	0	0.08
Kiss1r	0.005	0	0	0.03
EFHC1	0.005	0	0.005	0.005
RAB3IL1	0.005	0	0.005	0
CHRNA7	0.005	0	0.005	0
Chn2	0.005	0	0.005	0
Bach1	0	0	0.005	0.005
mRpS33	0	0	0.005	0
FLJ46111	0	0	0.01	0
Cpz	0	0	0.01	0
