# Supplementary figures and images for: A predictive signature gene set for discriminating active from latent tuberculosis in Warao Amerindian children
Source: BMC Genomics. 2013 Feb 1;14:74. doi: 10.1186/1471-2164-14-74 (PMC3600014; doi:10.1186/1471-2164-14-74)

ACOT7 ( $p = 0.23$ )

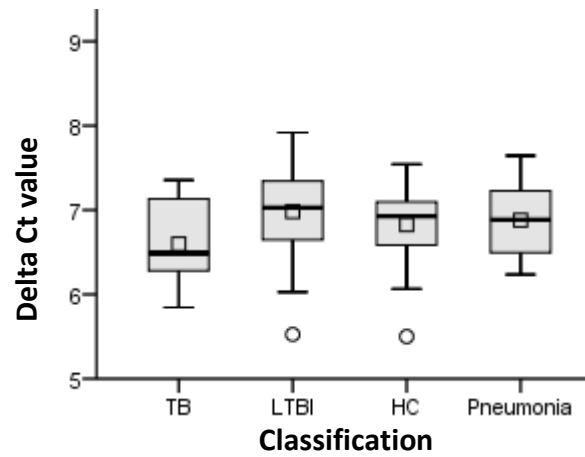

AMPH ( $p = 0.96$ )

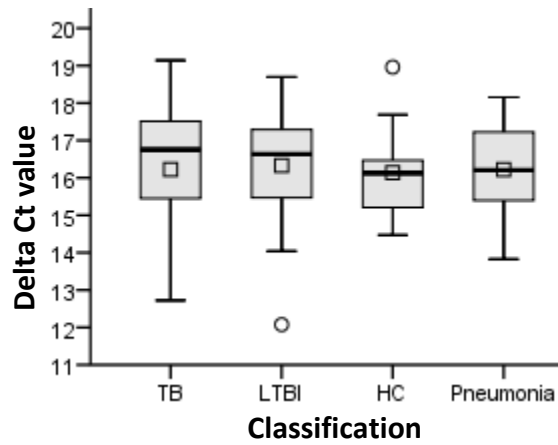

CHRM2 ( $p = 0.29$ )

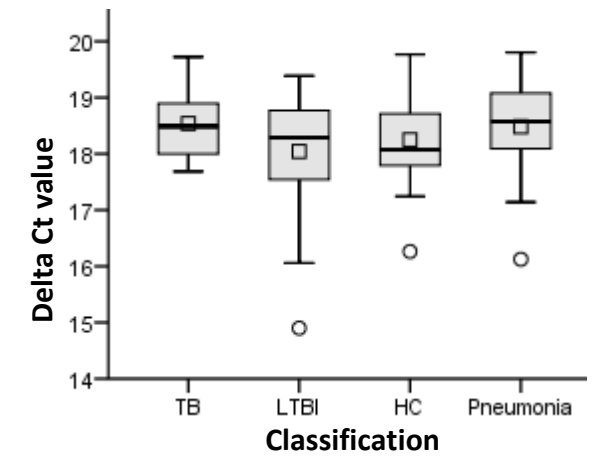

GLDC ( $p = 0.016$ )

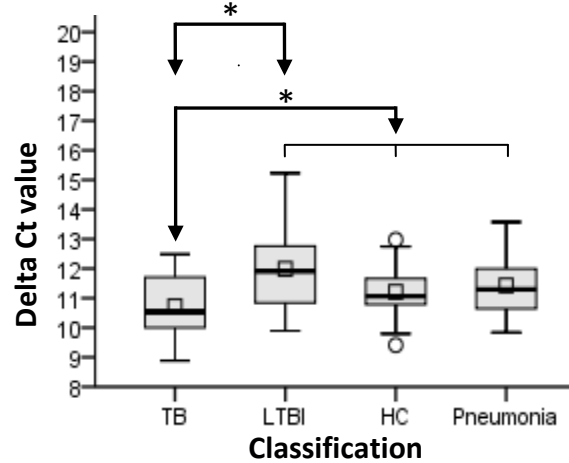

HBD ( $p < 0.01$ )

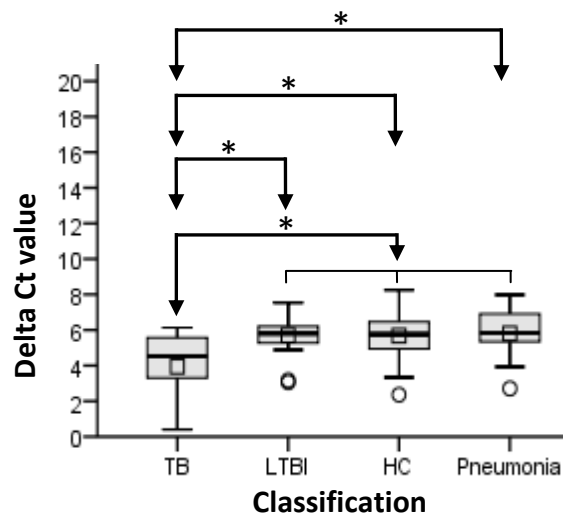

PIGC ( $p = 0.021$ )

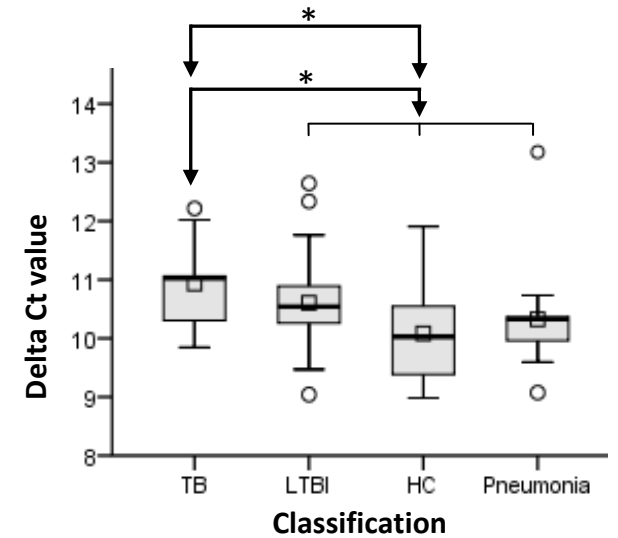

S100P ( $p < 0.01$ )

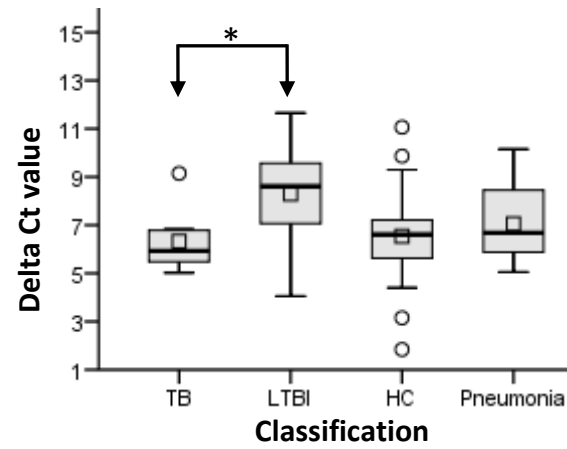

SNX17 ( $p = 0.032$ )

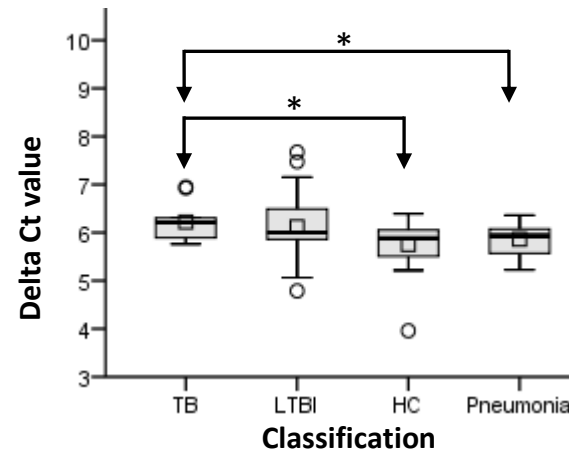

STYXL1 ( $p = 0.14$ )

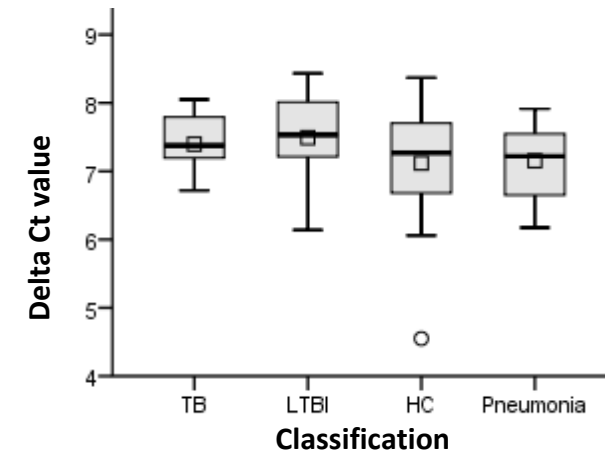

TAS2R46 ( $p = 0.096$ )

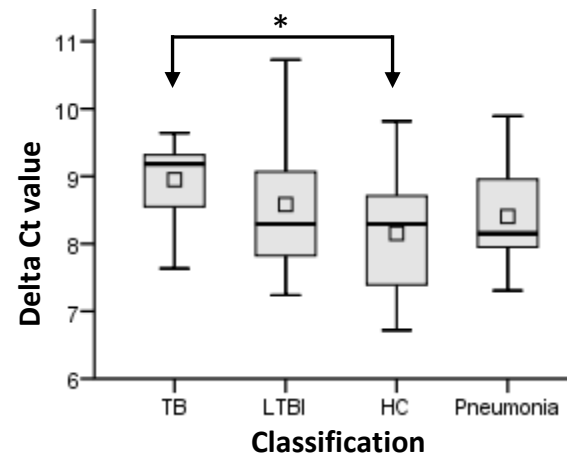

Supplement: Additional file 4: Figure S1 — qRT-PCR cycle threshold (delta Ct) values in TB, LTBI, HC and non-TB pneumonia subjects for each of the ten signature genes. The delta Ct was calculated as Ct value (number of cycles required for the fluorescent signal to exceed the background level) of the target gene – Ct value of the reference gene (GAPDH). The boxplots show the median delta Ct values and the interquartile ranges. The whiskers represent the highest and lowest values that are not outliers. Dots represent outliers and squares in the boxes indicate mean delta Ct values. The p-values for each gene (in parentheses) are the outcomes of the one-way analyses of variance comparing TB, LTBI, HC and non-TB pneumonia. The asterisks indicate statistically significant differences (p < 0.05) between TB and other groups as found by an unpaired Student’s t test. [file 1471-2164-14-74-S4.pdf]
