# Supplementary material for: A predictive signature gene set for discriminating active from latent tuberculosis in Warao Amerindian children
Source: BMC Genomics. 2013 Feb 1;14:74. doi: 10.1186/1471-2164-14-74 (PMC3600014; doi:10.1186/1471-2164-14-74)
Supplement: Additional file 5: Table S4 — qRT-PCR cycle threshold (delta Ct) values in TB, LTBI, HC and non-TB pneumonia subjects for each of the ten signature genes. The delta Ct was calculated as Ct value (number of cycles required for the fluorescent signal to exceed the background level, a lower Delta Ct value indicates a higher expression) of the target gene – Ct value of the reference gene (GAPDH). [file 1471-2164-14-74-S5.pdf]

Classification	Gene	Subject	Delta Ct value
TB	ACOT7	TB1	6,28
TB	ACOT7	TB2	6,52
TB	ACOT7	TB3	6,49
TB	ACOT7	TB4	5,85
TB	ACOT7	TB5	6,32
TB	ACOT7	TB6	6,12
TB	ACOT7	TB7	7,32
TB	ACOT7	TB8	7,36
TB	ACOT7	TB9	7,13
TB	AMPH	TB1	12,72
TB	AMPH	TB2	17,65
TB	AMPH	TB3	19,14
TB	AMPH	TB4	16,76
TB	AMPH	TB5	17,51
TB	AMPH	TB6	13,15
TB	AMPH	TB7	16,24
TB	AMPH	TB8	17,39
TB	AMPH	TB9	15,45
TB	CHRM2	TB1	17,69
TB	CHRM2	TB2	18,00
TB	CHRM2	TB3	19,14
TB	CHRM2	TB4	18,49
TB	CHRM2	TB5	18,89
TB	CHRM2	TB6	17,99
TB	CHRM2	TB7	18,78
TB	CHRM2	TB8	18,15
TB	CHRM2	TB9	19,72
TB	GLDC	TB1	9,99
TB	GLDC	TB2	10,47
TB	GLDC	TB3	11,72
TB	GLDC	TB4	8,89
TB	GLDC	TB5	10,00
TB	GLDC	TB6	10,72
TB	GLDC	TB7	10,53
TB	GLDC	TB8	12,48
TB	GLDC	TB9	11,71
TB	HBD	TB1	1,68
TB	HBD	TB2	3,43
TB	HBD	TB3	5,57
TB	HBD	TB4	4,64
TB	HBD	TB5	3,30
TB	HBD	TB6	0,41
TB	HBD	TB7	4,53
TB	HBD	TB8	6,14
TB	HBD	TB9	5,79
TB	PIGC	TB1	10,29
TB	PIGC	TB2	11,03
TB	PIGC	TB3	10,30
TB	PIGC	TB4	10,62
TB	PIGC	TB5	11,06
TB	PIGC	TB6	12,02
TB	PIGC	TB7	9,84
TB	PIGC	TB8	11,03
TB	PIGC	TB9	12,22
TB	S100P	TB1	9,15
TB	S100P	TB2	5,48
TB	S100P	TB3	5,93
TB	S100P	TB4	6,78
TB	S100P	TB5	6,83
TB	S100P	TB6	6,74
TB	S100P	TB7	5,11
TB	S100P	TB8	5,03
TB	S100P	TB9	5,90
TB	SNX17	TB1	5,83
TB	SNX17	TB2	5,89
TB	SNX17	TB3	6,30
TB	SNX17	TB4	6,21
TB	SNX17	TB5	5,76
TB	SNX17	TB6	6,93
TB	SNX17	TB7	5,90
TB	SNX17	TB8	6,22
TB	SNX17	TB9	6,95
TB	STYXL1	TB1	7,80
TB	STYXL1	TB2	6,74
TB	STYXL1	TB3	7,38
TB	STYXL1	TB4	8,05
TB	STYXL1	TB5	7,53
TB	STYXL1	TB6	7,85
TB	STYXL1	TB7	7,19
TB	STYXL1	TB8	7,32
TB	STYXL1	TB9	6,72
TB	TAS2R46	TB1	8,27
TB	TAS2R46	TB2	9,64
TB	TAS2R46	TB3	9,05
TB	TAS2R46	TB4	8,55
TB	TAS2R46	TB5	9,18
TB	TAS2R46	TB6	9,32
TB	TAS2R46	TB7	9,28
TB	TAS2R46	TB8	7,64
TB	TAS2R46	TB9	9,62
HC	ACOT7	HC1	7,48
HC	ACOT7	HC2	6,64
HC	ACOT7	HC3	7,29
HC	ACOT7	HC4	7,07
HC	ACOT7	HC5	6,97
HC	ACOT7	HC6	6,25
HC	ACOT7	HC7	6,93
HC	ACOT7	HC8	7,54
HC	ACOT7	HC9	6,94
HC	ACOT7	HC10	6,07
HC	ACOT7	HC11	7,22
HC	ACOT7	HC12	6,81
HC	ACOT7	HC13	6,92
HC	ACOT7	HC14	6,33
HC	ACOT7	HC15	6,99
HC	ACOT7	HC16	6,47
HC	ACOT7	HC17	5,50
HC	ACOT7	HC18	6,58
HC	ACOT7	HC19	6,74
HC	ACOT7	HC20	6,93
HC	ACOT7	HC21	7,10
HC	ACOT7	HC22	7,35
HC	ACOT7	HC23	6,50
HC	ACOT7	HC24	7,17
HC	ACOT7	HC25	6,90
HC	AMPH	HC1	15,78
HC	AMPH	HC2	16,72
HC	AMPH	HC3	16,13
HC	AMPH	HC4	18,96
HC	AMPH	HC5	14,79
HC	AMPH	HC6	16,47
HC	AMPH	HC7	15,21
HC	AMPH	HC8	16,04
HC	AMPH	HC9	16,17
HC	AMPH	HC10	17,65
HC	AMPH	HC11	15,19
HC	AMPH	HC12	14,47
HC	AMPH	HC13	16,19
HC	AMPH	HC14	16,05
HC	AMPH	HC15	16,46
HC	AMPH	HC16	17,69
HC	AMPH	HC17	14,74
HC	AMPH	HC18	17,31
HC	AMPH	HC19	16,15
HC	AMPH	HC20	16,38
HC	AMPH	HC21	15,75
HC	AMPH	HC22	14,94
HC	AMPH	HC23	17,19
HC	AMPH	HC24	15,88
HC	AMPH	HC25	15,20
HC	CHRM2	HC1	17,79
HC	CHRM2	HC2	17,92
HC	CHRM2	HC3	18,64
HC	CHRM2	HC4	18,05
HC	CHRM2	HC5	19,77
HC	CHRM2	HC6	18,08
HC	CHRM2	HC7	17,62
HC	CHRM2	HC8	19,09
HC	CHRM2	HC9	18,69
HC	CHRM2	HC10	18,60
HC	CHRM2	HC11	17,98
HC	CHRM2	HC12	18,71
HC	CHRM2	HC13	18,16
HC	CHRM2	HC14	17,57
HC	CHRM2	HC15	17,92
HC	CHRM2	HC16	18,52
HC	CHRM2	HC17	16,26
HC	CHRM2	HC18	18,01
HC	CHRM2	HC19	17,65
HC	CHRM2	HC20	19,61
HC	CHRM2	HC21	19,10
HC	CHRM2	HC22	17,25
HC	CHRM2	HC23	18,76
HC	CHRM2	HC24	17,51
HC	CHRM2	HC25	18,98
HC	GLDC	HC1	11,75
HC	GLDC	HC2	10,86
HC	GLDC	HC3	10,88
HC	GLDC	HC4	11,59
HC	GLDC	HC5	11,09
HC	GLDC	HC6	10,79
HC	GLDC	HC7	11,02
HC	GLDC	HC8	12,28
HC	GLDC	HC9	11,66
HC	GLDC	HC10	11,42
HC	GLDC	HC11	11,06
HC	GLDC	HC12	12,74
HC	GLDC	HC13	9,80
HC	GLDC	HC14	11,18
HC	GLDC	HC15	10,44
HC	GLDC	HC16	10,46
HC	GLDC	HC17	9,41
HC	GLDC	HC18	10,51
HC	GLDC	HC19	10,93
HC	GLDC	HC20	11,64
HC	GLDC	HC21	12,11
HC	GLDC	HC22	10,95
HC	GLDC	HC23	10,06
HC	GLDC	HC24	12,98
HC	GLDC	HC25	12,67
HC	HBD	HC1	4,94
HC	HBD	HC2	6,23
HC	HBD	HC3	8,25
HC	HBD	HC4	7,52
HC	HBD	HC5	2,36
HC	HBD	HC6	5,00
HC	HBD	HC7	6,92
HC	HBD	HC8	6,11
HC	HBD	HC9	6,46
HC	HBD	HC10	5,76
HC	HBD	HC11	7,38
HC	HBD	HC12	6,38
HC	HBD	HC13	4,13
HC	HBD	HC14	6,43
HC	HBD	HC15	6,46
HC	HBD	HC16	5,59
HC	HBD	HC17	4,12
HC	HBD	HC18	5,10
HC	HBD	HC19	5,20
HC	HBD	HC20	3,33
HC	HBD	HC21	7,04
HC	HBD	HC22	6,79
HC	HBD	HC23	4,79
HC	HBD	HC24	5,23
HC	HBD	HC25	4,49
HC	PIGC	HC1	10,63
HC	PIGC	HC2	9,29
HC	PIGC	HC3	9,94
HC	PIGC	HC4	10,02
HC	PIGC	HC5	9,39
HC	PIGC	HC6	10,22
HC	PIGC	HC7	9,68
HC	PIGC	HC8	10,11
HC	PIGC	HC9	9,16
HC	PIGC	HC10	9,77
HC	PIGC	HC11	9,38
HC	PIGC	HC12	11,91
HC	PIGC	HC13	10,54
HC	PIGC	HC14	10,62
HC	PIGC	HC15	10,93
HC	PIGC	HC16	11,39
HC	PIGC	HC17	8,98
HC	PIGC	HC18	10,10
HC	PIGC	HC19	10,50
HC	PIGC	HC20	9,93
HC	PIGC	HC21	11,06
HC	PIGC	HC22	9,05
HC	PIGC	HC23	10,03
HC	PIGC	HC24	10,10
HC	PIGC	HC25	9,35
HC	S100P	HC1	3,15
HC	S100P	HC2	5,51
HC	S100P	HC3	6,68
HC	S100P	HC4	4,40
HC	S100P	HC5	6,03
HC	S100P	HC6	5,90
HC	S100P	HC7	4,97
HC	S100P	HC8	5,62
HC	S100P	HC9	7,20
HC	S100P	HC10	11,06
HC	S100P	HC11	6,88
HC	S100P	HC12	6,04
HC	S100P	HC13	7,21
HC	S100P	HC14	6,84
HC	S100P	HC15	7,81
HC	S100P	HC16	6,58
HC	S100P	HC17	1,84
HC	S100P	HC18	9,29
HC	S100P	HC19	8,34
HC	S100P	HC20	5,07
HC	S100P	HC21	6,61
HC	S100P	HC22	7,70
HC	S100P	HC23	9,86
HC	S100P	HC24	5,82
HC	S100P	HC25	7,05
HC	SNX17	HC1	5,88
HC	SNX17	HC2	5,21
HC	SNX17	HC3	5,69
HC	SNX17	HC4	5,48
HC	SNX17	HC5	6,08
HC	SNX17	HC6	5,62
HC	SNX17	HC7	6,02
HC	SNX17	HC8	5,61
HC	SNX17	HC9	5,21
HC	SNX17	HC10	5,44
HC	SNX17	HC11	6,20
HC	SNX17	HC12	6,28
HC	SNX17	HC13	5,83
HC	SNX17	HC14	6,05
HC	SNX17	HC15	6,36
HC	SNX17	HC16	6,39
HC	SNX17	HC17	3,96
HC	SNX17	HC18	5,96
HC	SNX17	HC19	5,93
HC	SNX17	HC20	5,93
HC	SNX17	HC21	6,08
HC	SNX17	HC22	5,40
HC	SNX17	HC23	5,51
HC	SNX17	HC24	5,68
HC	SNX17	HC25	6,02
HC	STYXL1	HC1	6,06
HC	STYXL1	HC2	6,46
HC	STYXL1	HC3	6,59
HC	STYXL1	HC4	7,38
HC	STYXL1	HC5	7,88
HC	STYXL1	HC6	7,24
HC	STYXL1	HC7	7,38
HC	STYXL1	HC8	6,68
HC	STYXL1	HC9	6,63
HC	STYXL1	HC10	7,70
HC	STYXL1	HC11	7,76
HC	STYXL1	HC12	7,50
HC	STYXL1	HC13	8,14
HC	STYXL1	HC14	6,98
HC	STYXL1	HC15	8,37
HC	STYXL1	HC16	7,50
HC	STYXL1	HC17	4,55
HC	STYXL1	HC18	7,88
HC	STYXL1	HC19	6,80
HC	STYXL1	HC20	7,32
HC	STYXL1	HC21	7,89
HC	STYXL1	HC22	6,98
HC	STYXL1	HC23	6,27
HC	STYXL1	HC24	6,76
HC	STYXL1	HC25	7,27
HC	TAS2R46	HC1	6,91
HC	TAS2R46	HC2	7,15
HC	TAS2R46	HC3	7,53
HC	TAS2R46	HC4	6,93
HC	TAS2R46	HC5	8,74
HC	TAS2R46	HC6	7,39
HC	TAS2R46	HC7	8,73
HC	TAS2R46	HC8	8,14
HC	TAS2R46	HC9	7,32
HC	TAS2R46	HC10	8,70
HC	TAS2R46	HC11	8,71
HC	TAS2R46	HC12	9,82
HC	TAS2R46	HC13	8,52
HC	TAS2R46	HC14	8,47
HC	TAS2R46	HC15	9,36
HC	TAS2R46	HC16	9,45
HC	TAS2R46	HC17	6,72
HC	TAS2R46	HC18	8,43
HC	TAS2R46	HC19	8,26
HC	TAS2R46	HC20	8,13
HC	TAS2R46	HC21	8,29
HC	TAS2R46	HC22	6,93
HC	TAS2R46	HC23	8,60
HC	TAS2R46	HC24	7,80
HC	TAS2R46	HC25	8,81
LTBI	ACOT7	LTBI1	6,03
LTBI	ACOT7	LTBI2	5,53
LTBI	ACOT7	LTBI3	7,01
LTBI	ACOT7	LTBI4	6,26
LTBI	ACOT7	LTBI5	6,57
LTBI	ACOT7	LTBI6	6,91
LTBI	ACOT7	LTBI7	6,61
LTBI	ACOT7	LTBI8	7,16
LTBI	ACOT7	LTBI9	7,49
LTBI	ACOT7	LTBI10	7,37
LTBI	ACOT7	LTBI11	6,82
LTBI	ACOT7	LTBI12	6,68
LTBI	ACOT7	LTBI13	6,83
LTBI	ACOT7	LTBI14	7,30
LTBI	ACOT7	LTBI15	7,39
LTBI	ACOT7	LTBI16	7,09
LTBI	ACOT7	LTBI17	7,11
LTBI	ACOT7	LTBI18	6,99
LTBI	ACOT7	LTBI19	7,28
LTBI	ACOT7	LTBI20	7,73
LTBI	ACOT7	LTBI21	7,92
LTBI	ACOT7	LTBI22	7,14
LTBI	ACOT7	LTBI23	6,52
LTBI	ACOT7	LTBI24	6,29
LTBI	ACOT7	LTBI25	7,03
LTBI	ACOT7	LTBI26	7,34
LTBI	ACOT7	LTBI27	7,83
LTBI	ACOT7	LTBI28	6,65
LTBI	ACOT7	LTBI29	7,59
LTBI	AMPH	LTBI1	17,65
LTBI	AMPH	LTBI2	16,63
LTBI	AMPH	LTBI3	15,38
LTBI	AMPH	LTBI4	16,54
LTBI	AMPH	LTBI5	16,99
LTBI	AMPH	LTBI6	15,75
LTBI	AMPH	LTBI7	17,48
LTBI	AMPH	LTBI8	15,33
LTBI	AMPH	LTBI9	17,32
LTBI	AMPH	LTBI10	16,95
LTBI	AMPH	LTBI11	15,59
LTBI	AMPH	LTBI12	17,15
LTBI	AMPH	LTBI13	17,29
LTBI	AMPH	LTBI14	15,08
LTBI	AMPH	LTBI15	15,93
LTBI	AMPH	LTBI16	16,98
LTBI	AMPH	LTBI17	16,96
LTBI	AMPH	LTBI18	18,70
LTBI	AMPH	LTBI19	12,08
LTBI	AMPH	LTBI20	17,57
LTBI	AMPH	LTBI21	17,11
LTBI	AMPH	LTBI22	15,47
LTBI	AMPH	LTBI23	15,12
LTBI	AMPH	LTBI24	14,66
LTBI	AMPH	LTBI25	14,04
LTBI	AMPH	LTBI26	16,55
LTBI	AMPH	LTBI27	17,44
LTBI	AMPH	LTBI28	17,59
LTBI	AMPH	LTBI29	16,26
LTBI	CHRM2	LTBI1	19,14
LTBI	CHRM2	LTBI2	17,89
LTBI	CHRM2	LTBI3	17,84
LTBI	CHRM2	LTBI4	16,06
LTBI	CHRM2	LTBI5	17,51
LTBI	CHRM2	LTBI6	19,39
LTBI	CHRM2	LTBI7	19,28
LTBI	CHRM2	LTBI8	17,31
LTBI	CHRM2	LTBI9	18,26
LTBI	CHRM2	LTBI10	18,47
LTBI	CHRM2	LTBI11	18,47
LTBI	CHRM2	LTBI12	18,20
LTBI	CHRM2	LTBI13	18,77
LTBI	CHRM2	LTBI14	18,93
LTBI	CHRM2	LTBI15	16,86
LTBI	CHRM2	LTBI16	18,93
LTBI	CHRM2	LTBI17	18,73
LTBI	CHRM2	LTBI18	16,85
LTBI	CHRM2	LTBI19	18,51
LTBI	CHRM2	LTBI20	18,52
LTBI	CHRM2	LTBI21	18,79
LTBI	CHRM2	LTBI22	17,88
LTBI	CHRM2	LTBI23	18,29
LTBI	CHRM2	LTBI24	18,78
LTBI	CHRM2	LTBI25	14,90
LTBI	CHRM2	LTBI26	17,22
LTBI	CHRM2	LTBI27	18,34
LTBI	CHRM2	LTBI28	17,59
LTBI	CHRM2	LTBI29	17,54
LTBI	GLDC	LTBI1	11,37
LTBI	GLDC	LTBI2	10,11
LTBI	GLDC	LTBI3	12,16
LTBI	GLDC	LTBI4	12,76
LTBI	GLDC	LTBI5	12,59
LTBI	GLDC	LTBI6	14,16
LTBI	GLDC	LTBI7	10,84
LTBI	GLDC	LTBI8	10,44
LTBI	GLDC	LTBI9	12,90
LTBI	GLDC	LTBI10	13,37
LTBI	GLDC	LTBI11	12,62
LTBI	GLDC	LTBI12	14,59
LTBI	GLDC	LTBI13	10,89
LTBI	GLDC	LTBI14	12,82
LTBI	GLDC	LTBI15	12,52
LTBI	GLDC	LTBI16	10,59
LTBI	GLDC	LTBI17	11,85
LTBI	GLDC	LTBI18	9,89
LTBI	GLDC	LTBI19	13,46
LTBI	GLDC	LTBI20	15,22
LTBI	GLDC	LTBI21	11,63
LTBI	GLDC	LTBI22	10,01
LTBI	GLDC	LTBI23	10,49
LTBI	GLDC	LTBI24	10,77
LTBI	GLDC	LTBI25	11,91
LTBI	GLDC	LTBI26	12,62
LTBI	GLDC	LTBI27	11,45
LTBI	GLDC	LTBI28	11,92
LTBI	GLDC	LTBI29	11,75
LTBI	HBD	LTBI1	6,18
LTBI	HBD	LTBI2	4,93
LTBI	HBD	LTBI3	5,26
LTBI	HBD	LTBI4	5,37
LTBI	HBD	LTBI5	5,86
LTBI	HBD	LTBI6	5,52
LTBI	HBD	LTBI7	6,07
LTBI	HBD	LTBI8	5,47
LTBI	HBD	LTBI9	6,09
LTBI	HBD	LTBI10	6,52
LTBI	HBD	LTBI11	7,10
LTBI	HBD	LTBI12	5,20
LTBI	HBD	LTBI13	3,06
LTBI	HBD	LTBI14	5,12
LTBI	HBD	LTBI15	5,81
LTBI	HBD	LTBI16	6,39
LTBI	HBD	LTBI17	7,53
LTBI	HBD	LTBI18	4,89
LTBI	HBD	LTBI19	6,82
LTBI	HBD	LTBI20	5,88
LTBI	HBD	LTBI21	5,26
LTBI	HBD	LTBI22	5,76
LTBI	HBD	LTBI23	5,64
LTBI	HBD	LTBI24	6,01
LTBI	HBD	LTBI25	6,20
LTBI	HBD	LTBI26	3,17
LTBI	HBD	LTBI27	6,52
LTBI	HBD	LTBI28	5,29
LTBI	HBD	LTBI29	6,39
LTBI	PIGC	LTBI1	9,58
LTBI	PIGC	LTBI2	9,53
LTBI	PIGC	LTBI3	10,44
LTBI	PIGC	LTBI4	10,30
LTBI	PIGC	LTBI5	10,32
LTBI	PIGC	LTBI6	10,46
LTBI	PIGC	LTBI7	10,74
LTBI	PIGC	LTBI8	10,43
LTBI	PIGC	LTBI9	10,27
LTBI	PIGC	LTBI10	10,58
LTBI	PIGC	LTBI11	10,54
LTBI	PIGC	LTBI12	10,14
LTBI	PIGC	LTBI13	10,74
LTBI	PIGC	LTBI14	10,89
LTBI	PIGC	LTBI15	12,64
LTBI	PIGC	LTBI16	9,04
LTBI	PIGC	LTBI17	10,56
LTBI	PIGC	LTBI18	10,04
LTBI	PIGC	LTBI19	10,26
LTBI	PIGC	LTBI20	10,84
LTBI	PIGC	LTBI21	11,58
LTBI	PIGC	LTBI22	9,83
LTBI	PIGC	LTBI23	10,88
LTBI	PIGC	LTBI24	9,47
LTBI	PIGC	LTBI25	11,76
LTBI	PIGC	LTBI26	12,34
LTBI	PIGC	LTBI27	11,14
LTBI	PIGC	LTBI28	11,12
LTBI	PIGC	LTBI29	11,12
LTBI	S100P	LTBI1	10,13
LTBI	S100P	LTBI2	7,86
LTBI	S100P	LTBI3	9,80
LTBI	S100P	LTBI4	5,78
LTBI	S100P	LTBI5	8,66
LTBI	S100P	LTBI6	9,79
LTBI	S100P	LTBI7	7,07
LTBI	S100P	LTBI8	10,95
LTBI	S100P	LTBI9	8,47
LTBI	S100P	LTBI10	9,09
LTBI	S100P	LTBI11	7,22
LTBI	S100P	LTBI12	9,78
LTBI	S100P	LTBI13	10,01
LTBI	S100P	LTBI14	11,65
LTBI	S100P	LTBI15	8,29
LTBI	S100P	LTBI16	8,61
LTBI	S100P	LTBI17	9,57
LTBI	S100P	LTBI18	6,80
LTBI	S100P	LTBI19	9,07
LTBI	S100P	LTBI20	5,79
LTBI	S100P	LTBI21	8,96
LTBI	S100P	LTBI22	8,52
LTBI	S100P	LTBI23	8,99
LTBI	S100P	LTBI24	5,86
LTBI	S100P	LTBI25	5,35
LTBI	S100P	LTBI26	6,71
LTBI	S100P	LTBI27	4,05
LTBI	S100P	LTBI28	9,55
LTBI	S100P	LTBI29	8,11
LTBI	SNX17	LTBI1	5,86
LTBI	SNX17	LTBI2	4,79
LTBI	SNX17	LTBI3	6,54
LTBI	SNX17	LTBI4	5,18
LTBI	SNX17	LTBI5	5,76
LTBI	SNX17	LTBI6	6,00
LTBI	SNX17	LTBI7	6,49
LTBI	SNX17	LTBI8	6,01
LTBI	SNX17	LTBI9	6,31
LTBI	SNX17	LTBI10	7,68
LTBI	SNX17	LTBI11	7,47
LTBI	SNX17	LTBI12	7,16
LTBI	SNX17	LTBI13	5,90
LTBI	SNX17	LTBI14	6,18
LTBI	SNX17	LTBI15	5,89
LTBI	SNX17	LTBI16	5,06
LTBI	SNX17	LTBI17	5,85
LTBI	SNX17	LTBI18	5,98
LTBI	SNX17	LTBI19	6,22
LTBI	SNX17	LTBI20	6,99
LTBI	SNX17	LTBI21	6,51
LTBI	SNX17	LTBI22	5,73
LTBI	SNX17	LTBI23	5,94
LTBI	SNX17	LTBI24	5,21
LTBI	SNX17	LTBI25	5,96
LTBI	SNX17	LTBI26	6,14
LTBI	SNX17	LTBI27	6,69
LTBI	SNX17	LTBI28	5,87
LTBI	SNX17	LTBI29	6,11
LTBI	STYXL1	LTBI1	7,28
LTBI	STYXL1	LTBI2	6,14
LTBI	STYXL1	LTBI3	8,44
LTBI	STYXL1	LTBI4	7,90
LTBI	STYXL1	LTBI5	6,72
LTBI	STYXL1	LTBI6	7,21
LTBI	STYXL1	LTBI7	7,65
LTBI	STYXL1	LTBI8	7,49
LTBI	STYXL1	LTBI9	7,55
LTBI	STYXL1	LTBI10	7,52
LTBI	STYXL1	LTBI11	8,15
LTBI	STYXL1	LTBI12	7,47
LTBI	STYXL1	LTBI13	7,95
LTBI	STYXL1	LTBI14	8,02
LTBI	STYXL1	LTBI15	6,98
LTBI	STYXL1	LTBI16	6,43
LTBI	STYXL1	LTBI17	7,67
LTBI	STYXL1	LTBI18	6,53
LTBI	STYXL1	LTBI19	7,39
LTBI	STYXL1	LTBI20	7,84
LTBI	STYXL1	LTBI21	8,04
LTBI	STYXL1	LTBI22	8,24
LTBI	STYXL1	LTBI23	7,21
LTBI	STYXL1	LTBI24	6,47
LTBI	STYXL1	LTBI25	7,53
LTBI	STYXL1	LTBI26	8,01
LTBI	STYXL1	LTBI27	8,24
LTBI	STYXL1	LTBI28	7,15
LTBI	STYXL1	LTBI29	8,05
LTBI	TAS2R46	LTBI1	7,99
LTBI	TAS2R46	LTBI2	7,38
LTBI	TAS2R46	LTBI3	9,35
LTBI	TAS2R46	LTBI4	7,61
LTBI	TAS2R46	LTBI5	7,63
LTBI	TAS2R46	LTBI6	8,49
LTBI	TAS2R46	LTBI7	9,89
LTBI	TAS2R46	LTBI8	8,81
LTBI	TAS2R46	LTBI9	8,05
LTBI	TAS2R46	LTBI10	9,96
LTBI	TAS2R46	LTBI11	8,97
LTBI	TAS2R46	LTBI12	8,83
LTBI	TAS2R46	LTBI13	9,07
LTBI	TAS2R46	LTBI14	8,73
LTBI	TAS2R46	LTBI15	10,73
LTBI	TAS2R46	LTBI16	7,40
LTBI	TAS2R46	LTBI17	8,16
LTBI	TAS2R46	LTBI18	8,63
LTBI	TAS2R46	LTBI19	7,98
LTBI	TAS2R46	LTBI20	7,69
LTBI	TAS2R46	LTBI21	9,62
LTBI	TAS2R46	LTBI22	8,29
LTBI	TAS2R46	LTBI23	8,05
LTBI	TAS2R46	LTBI24	7,24
LTBI	TAS2R46	LTBI25	10,48
LTBI	TAS2R46	LTBI26	7,83
LTBI	TAS2R46	LTBI27	8,23
LTBI	TAS2R46	LTBI28	7,39
LTBI	TAS2R46	LTBI29	10,40
Non-TB pneumonia	ACOT7	PNEU1	6,99
Non-TB pneumonia	ACOT7	PNEU2	6,70
Non-TB pneumonia	ACOT7	PNEU3	7,23
Non-TB pneumonia	ACOT7	PNEU4	6,63
Non-TB pneumonia	ACOT7	PNEU5	6,54
Non-TB pneumonia	ACOT7	PNEU6	6,46
Non-TB pneumonia	ACOT7	PNEU7	6,44
Non-TB pneumonia	ACOT7	PNEU8	6,78
Non-TB pneumonia	ACOT7	PNEU9	6,24
Non-TB pneumonia	ACOT7	PNEU10	6,99
Non-TB pneumonia	ACOT7	PNEU11	7,64
Non-TB pneumonia	ACOT7	PNEU12	7,07
Non-TB pneumonia	ACOT7	PNEU13	7,39
Non-TB pneumonia	ACOT7	PNEU14	7,40
Non-TB pneumonia	ACOT7	PNEU15	6,39
Non-TB pneumonia	ACOT7	PNEU16	7,30
Non-TB pneumonia	ACOT7	PNEU17	7,16
Non-TB pneumonia	ACOT7	PNEU18	6,50
Non-TB pneumonia	AMPH	PNEU1	16,58
Non-TB pneumonia	AMPH	PNEU2	17,00
Non-TB pneumonia	AMPH	PNEU3	18,16
Non-TB pneumonia	AMPH	PNEU4	17,04
Non-TB pneumonia	AMPH	PNEU5	17,25
Non-TB pneumonia	AMPH	PNEU6	15,95
Non-TB pneumonia	AMPH	PNEU7	13,83
Non-TB pneumonia	AMPH	PNEU8	14,54
Non-TB pneumonia	AMPH	PNEU9	15,60
Non-TB pneumonia	AMPH	PNEU10	15,68
Non-TB pneumonia	AMPH	PNEU11	18,11
Non-TB pneumonia	AMPH	PNEU12	16,46
Non-TB pneumonia	AMPH	PNEU13	17,50
Non-TB pneumonia	AMPH	PNEU14	15,16
Non-TB pneumonia	AMPH	PNEU15	15,09
Non-TB pneumonia	AMPH	PNEU16	17,22
Non-TB pneumonia	AMPH	PNEU17	15,43
Non-TB pneumonia	AMPH	PNEU18	15,39
Non-TB pneumonia	CHRM2	PNEU1	17,14
Non-TB pneumonia	CHRM2	PNEU2	18,57
Non-TB pneumonia	CHRM2	PNEU3	18,16
Non-TB pneumonia	CHRM2	PNEU4	18,04
Non-TB pneumonia	CHRM2	PNEU5	16,13
Non-TB pneumonia	CHRM2	PNEU6	18,29
Non-TB pneumonia	CHRM2	PNEU7	17,82
Non-TB pneumonia	CHRM2	PNEU8	19,80
Non-TB pneumonia	CHRM2	PNEU9	19,08
Non-TB pneumonia	CHRM2	PNEU10	19,48
Non-TB pneumonia	CHRM2	PNEU11	19,48
Non-TB pneumonia	CHRM2	PNEU12	18,58
Non-TB pneumonia	CHRM2	PNEU13	18,82
Non-TB pneumonia	CHRM2	PNEU14	19,47
Non-TB pneumonia	CHRM2	PNEU15	18,37
Non-TB pneumonia	CHRM2	PNEU16	18,72
Non-TB pneumonia	CHRM2	PNEU17	18,09
Non-TB pneumonia	CHRM2	PNEU18	18,66
Non-TB pneumonia	GLDC	PNEU1	13,57
Non-TB pneumonia	GLDC	PNEU2	9,89
Non-TB pneumonia	GLDC	PNEU3	13,55
Non-TB pneumonia	GLDC	PNEU4	10,92
Non-TB pneumonia	GLDC	PNEU5	12,27
Non-TB pneumonia	GLDC	PNEU6	11,27
Non-TB pneumonia	GLDC	PNEU7	9,84
Non-TB pneumonia	GLDC	PNEU8	10,13
Non-TB pneumonia	GLDC	PNEU9	12,48
Non-TB pneumonia	GLDC	PNEU10	10,29
Non-TB pneumonia	GLDC	PNEU11	11,30
Non-TB pneumonia	GLDC	PNEU12	10,64
Non-TB pneumonia	GLDC	PNEU13	11,99
Non-TB pneumonia	GLDC	PNEU14	11,29
Non-TB pneumonia	GLDC	PNEU15	11,76
Non-TB pneumonia	GLDC	PNEU16	11,32
Non-TB pneumonia	GLDC	PNEU17	11,22
Non-TB pneumonia	GLDC	PNEU18	11,96
Non-TB pneumonia	HBD	PNEU1	7,05
Non-TB pneumonia	HBD	PNEU2	7,06
Non-TB pneumonia	HBD	PNEU3	5,58
Non-TB pneumonia	HBD	PNEU4	5,43
Non-TB pneumonia	HBD	PNEU5	6,38
Non-TB pneumonia	HBD	PNEU6	6,21
Non-TB pneumonia	HBD	PNEU7	2,71
Non-TB pneumonia	HBD	PNEU8	5,90
Non-TB pneumonia	HBD	PNEU9	7,98
Non-TB pneumonia	HBD	PNEU10	5,76
Non-TB pneumonia	HBD	PNEU11	6,33
Non-TB pneumonia	HBD	PNEU12	6,90
Non-TB pneumonia	HBD	PNEU13	4,90
Non-TB pneumonia	HBD	PNEU14	5,35
Non-TB pneumonia	HBD	PNEU15	3,93
Non-TB pneumonia	HBD	PNEU16	5,52
Non-TB pneumonia	HBD	PNEU17	7,15
Non-TB pneumonia	HBD	PNEU18	4,29
Non-TB pneumonia	PIGC	PNEU1	10,17
Non-TB pneumonia	PIGC	PNEU2	10,33
Non-TB pneumonia	PIGC	PNEU3	10,35
Non-TB pneumonia	PIGC	PNEU4	10,36
Non-TB pneumonia	PIGC	PNEU5	10,73
Non-TB pneumonia	PIGC	PNEU6	10,22
Non-TB pneumonia	PIGC	PNEU7	10,31
Non-TB pneumonia	PIGC	PNEU8	10,64
Non-TB pneumonia	PIGC	PNEU9	9,07
Non-TB pneumonia	PIGC	PNEU10	10,44
Non-TB pneumonia	PIGC	PNEU11	10,37
Non-TB pneumonia	PIGC	PNEU12	10,18
Non-TB pneumonia	PIGC	PNEU13	9,96
Non-TB pneumonia	PIGC	PNEU14	9,60
Non-TB pneumonia	PIGC	PNEU15	10,36
Non-TB pneumonia	PIGC	PNEU16	9,92
Non-TB pneumonia	PIGC	PNEU17	9,66
Non-TB pneumonia	PIGC	PNEU18	13,18
Non-TB pneumonia	S100P	PNEU1	6,36
Non-TB pneumonia	S100P	PNEU2	7,83
Non-TB pneumonia	S100P	PNEU3	10,15
Non-TB pneumonia	S100P	PNEU4	5,88
Non-TB pneumonia	S100P	PNEU5	6,70
Non-TB pneumonia	S100P	PNEU6	8,89
Non-TB pneumonia	S100P	PNEU7	8,45
Non-TB pneumonia	S100P	PNEU8	7,45
Non-TB pneumonia	S100P	PNEU9	5,13
Non-TB pneumonia	S100P	PNEU10	5,05
Non-TB pneumonia	S100P	PNEU11	9,17
Non-TB pneumonia	S100P	PNEU12	5,99
Non-TB pneumonia	S100P	PNEU13	6,09
Non-TB pneumonia	S100P	PNEU14	5,64
Non-TB pneumonia	S100P	PNEU15	9,33
Non-TB pneumonia	S100P	PNEU16	6,86
Non-TB pneumonia	S100P	PNEU17	6,66
Non-TB pneumonia	S100P	PNEU18	5,33
Non-TB pneumonia	SNX17	PNEU1	5,47
Non-TB pneumonia	SNX17	PNEU2	5,88
Non-TB pneumonia	SNX17	PNEU3	6,33
Non-TB pneumonia	SNX17	PNEU4	5,48
Non-TB pneumonia	SNX17	PNEU5	5,99
Non-TB pneumonia	SNX17	PNEU6	6,03
Non-TB pneumonia	SNX17	PNEU7	6,05
Non-TB pneumonia	SNX17	PNEU8	6,26
Non-TB pneumonia	SNX17	PNEU9	5,23
Non-TB pneumonia	SNX17	PNEU10	6,16
Non-TB pneumonia	SNX17	PNEU11	5,99
Non-TB pneumonia	SNX17	PNEU12	5,77
Non-TB pneumonia	SNX17	PNEU13	5,43
Non-TB pneumonia	SNX17	PNEU14	5,57
Non-TB pneumonia	SNX17	PNEU15	5,75
Non-TB pneumonia	SNX17	PNEU16	5,68
Non-TB pneumonia	SNX17	PNEU17	6,08
Non-TB pneumonia	SNX17	PNEU18	6,36
Non-TB pneumonia	STYXL1	PNEU1	7,82
Non-TB pneumonia	STYXL1	PNEU2	7,88
Non-TB pneumonia	STYXL1	PNEU3	7,91
Non-TB pneumonia	STYXL1	PNEU4	7,55
Non-TB pneumonia	STYXL1	PNEU5	7,73
Non-TB pneumonia	STYXL1	PNEU6	7,03
Non-TB pneumonia	STYXL1	PNEU7	7,50
Non-TB pneumonia	STYXL1	PNEU8	6,47
Non-TB pneumonia	STYXL1	PNEU9	6,78
Non-TB pneumonia	STYXL1	PNEU10	7,46
Non-TB pneumonia	STYXL1	PNEU11	6,65
Non-TB pneumonia	STYXL1	PNEU12	6,88
Non-TB pneumonia	STYXL1	PNEU13	6,59
Non-TB pneumonia	STYXL1	PNEU14	7,49
Non-TB pneumonia	STYXL1	PNEU15	6,17
Non-TB pneumonia	STYXL1	PNEU16	7,21
Non-TB pneumonia	STYXL1	PNEU17	6,41
Non-TB pneumonia	STYXL1	PNEU18	7,22
Non-TB pneumonia	TAS2R46	PNEU1	7,59
Non-TB pneumonia	TAS2R46	PNEU2	8,95
Non-TB pneumonia	TAS2R46	PNEU3	7,92
Non-TB pneumonia	TAS2R46	PNEU4	7,95
Non-TB pneumonia	TAS2R46	PNEU5	8,14
Non-TB pneumonia	TAS2R46	PNEU6	9,69
Non-TB pneumonia	TAS2R46	PNEU7	9,05
Non-TB pneumonia	TAS2R46	PNEU8	8,72
Non-TB pneumonia	TAS2R46	PNEU9	7,31
Non-TB pneumonia	TAS2R46	PNEU10	7,95
Non-TB pneumonia	TAS2R46	PNEU11	8,10
Non-TB pneumonia	TAS2R46	PNEU12	7,50
Non-TB pneumonia	TAS2R46	PNEU13	8,16
Non-TB pneumonia	TAS2R46	PNEU14	8,02
Non-TB pneumonia	TAS2R46	PNEU15	8,67
Non-TB pneumonia	TAS2R46	PNEU16	9,03
Non-TB pneumonia	TAS2R46	PNEU17	8,63
Non-TB pneumonia	TAS2R46	PNEU18	9,90
TB, recovery	ACOT7	TB3	7,57
TB, recovery	ACOT7	TB4	7,33
TB, recovery	ACOT7	TB9	6,91
TB, recovery	AMPH	TB3	15,85
TB, recovery	AMPH	TB4	14,99
TB, recovery	AMPH	TB9	18,76
TB, recovery	CHRM2	TB3	18,67
TB, recovery	CHRM2	TB4	17,91
TB, recovery	CHRM2	TB9	18,42
TB, recovery	GLDC	TB3	11,77
TB, recovery	GLDC	TB4	9,60
TB, recovery	GLDC	TB9	10,25
TB, recovery	HBD	TB3	5,61
TB, recovery	HBD	TB4	4,64
TB, recovery	HBD	TB9	8,01
TB, recovery	PIGC	TB3	10,58
TB, recovery	PIGC	TB4	9,95
TB, recovery	PIGC	TB9	10,67
TB, recovery	S100P	TB3	6,22
TB, recovery	S100P	TB4	6,92
TB, recovery	S100P	TB9	7,40
TB, recovery	SNX17	TB3	6,90
TB, recovery	SNX17	TB4	5,98
TB, recovery	SNX17	TB9	5,90
TB, recovery	STYXL1	TB3	7,56
TB, recovery	STYXL1	TB4	8,62
TB, recovery	STYXL1	TB9	7,14
TB, recovery	TAS2R46	TB3	8,82
TB, recovery	TAS2R46	TB4	9,93
TB, recovery	TAS2R46	TB9	8,86
